# Unique Molecular Identifiers reveal a novel sequencing artefact with implications for RNA-Seq based gene expression analysis

**DOI:** 10.1038/s41598-018-31064-7

**Published:** 2018-09-03

**Authors:** Johnny A. Sena, Giulia Galotto, Nico P. Devitt, Melanie C. Connick, Jennifer L. Jacobi, Pooja E. Umale, Luis Vidali, Callum J. Bell

**Affiliations:** 1 National Center for Genome Resources, Santa Fe, NM 87505 United States; 2 Worcester Polytechnic Institute, Department of Biology and Biotechnology, Worcester, MA 01609 United States

## Abstract

Attaching Unique Molecular Identifiers (UMI) to RNA molecules in the first step of sequencing library preparation establishes a distinct identity for each input molecule. This makes it possible to eliminate the effects of PCR amplification bias, which is particularly important where many PCR cycles are required, for example, in single cell studies. After PCR, molecules sharing a UMI are assumed to be derived from the same input molecule. In our single cell RNA-Seq studies of *Physcomitrella* *patens*, we discovered that reads sharing a UMI, and therefore presumed to be derived from the same mRNA molecule, frequently map to different, but closely spaced locations. This behaviour occurs in all such libraries that we have produced, and in multiple other UMI-containing RNA-Seq data sets in the public domain. This apparent paradox, that reads of identical origin map to distinct genomic coordinates may be partially explained by PCR stutter, which is often seen in low-entropy templates and those containing simple tandem repeats. In the absence of UMI this artefact is undetectable. We show that the common assumption that sequence reads having different mapping coordinates are derived from different starting molecules does not hold. Unless taken into account, this artefact is likely to result in over-estimation of certain transcript abundances, depending on the counting method employed.

## Introduction

Attaching random oligomers to DNA and RNA molecules before PCR amplification in order to detect contamination and to correct for amplification bias, particularly with low input samples, pre-dates next generation sequencing (NGS)^1,2^. More recently, inclusion of these Unique Molecular Identifiers (UMI) in the first steps of sequencing library preparation has become increasingly popular, and offers several benefits^3–5^. UMI create a distinct identity for each input molecule; this makes it possible to estimate the efficiency with which input molecules are sampled, identify sampling bias, and most importantly, identify and correct for the effects of PCR amplification bias. The last item is particularly important where many PCR cycles are required to achieve sufficient material to prepare the sample for sequencing, for example, in single cell studies. After PCR, molecules sharing a UMI are assumed to be derived from the same input molecule. UMI were used to investigate and model the nature of PCR amplification bias itself^6^, revealing read families representing single starting molecules spanning two orders of magnitude. This has had significant impact on transcriptomics studies, in which UMI counts per gene offer superior results to counting reads, and therefore provide more accurate estimates of quantitative gene expression^3,5^. UMI counting is the basis of quantitative gene expression profiling in the 10X Genomics Chromium single cell analysis system^7^. Kou *et al*.^4^ showed that although UMI provide effective correction for PCR amplification bias, the main source of bias in the relative representation of input molecules in post-PCR data is due to uneven sampling of starting template by RT or PCR primers. Unique Molecular Identifiers have also found utility in genomic DNA sequencing applications and in virology, where they assist with distinguishing rare sequence variants from reverse transcriptase, PCR and sequencing errors^8,9^. Some software tools for the analysis of transcriptome data, containing UMI, were recently made available^10^.

We are developing targeted RNA-Seq methods in order to study tip growth and other phenomena in *Physcomitrella patens* with single cell precision. And, in the course of these studies we observed that a considerable fraction of sequence reads, having the same UMI, map to different but closely spaced locations. For a given UMI, these locations form clusters, such that the majority of reads map to one location, with smaller numbers mapping closely upstream and downstream, forming a bell-shaped distribution. We call a group of closely spaced reads sharing a UMI a UMI-read cluster, or, for simplicity, just a cluster. The size of a UMI-read cluster is the number of adjacent coordinates sharing the UMI. This artefact is seen in all libraries containing UMI that we have made, and in all UMI-containing RNA-Seq data sets we have collected from the public domain. This is an apparent paradox, in which reads originating from the same template have overlapping sequences that map to adjacent locations. In this work, we characterize the nature of the artefact and its potential impact on analyses that depend on UMI-counting methods, and we describe the nature of reads and UMI that are associated with the phenomenon. We observed that sequences demonstrating the read mapping shift artefact tend to contain simple tandem repeats and that information theory metrics predictive of such repeats also predict the occurrence of the artefact, but not in all data sets. Accordingly, we conclude that PCR stutter, associated with low-entropy reads and those containing simple tandem repeats contributes to, but can only partially explain this artefact.

## Results

### Reads mapping to adjacent locations have the same UMI

We prepared multiple instances of Illumina RNA-Seq libraries using a modification of the protocol of Islam *et al*.^3^, in which the primers were changed to be compatible with Illumina paired end flowcells and the UMI consisted of a fully random 10-mer. Input material for library preparation was either a single *Physcomitrella patens* protoplast or a single-cell quantity of total RNA (approximately 23 pg). All libraries included PCR steps totaling 37 cycles. We pooled, sequenced and analysed these in two batches, run_170420 and run_171108. After removal of the UMI and further trimming (see Methods), 36 bp reads were aligned to the *Physcomitrella patens* genome^11^. Only reads aligning to a single location in the genome were studied further. Run_171108 contained data from 36 libraries made from single cells or cell equivalents, consisting of 158,663,380 uniquely aligning reads. Run_170420 contained three single cell equivalents and three 10 cell equivalents consisting of 49,729,975 uniquely aligning reads. Six bp indexes were applied to each library to allow demultiplexing and study of libraries individually. When biological replicates were examined (two protoplasts), they showed the characteristic improvement in dispersion when UMI aligning to genes are counted as opposed to reads, an example being shown in Supplementary Fig. S1. Because we are concerned here with the alignment characteristics of these reads with respect to their UMI, further analysis was done on pooled data without regard to library indexes.

**Figure 1:**
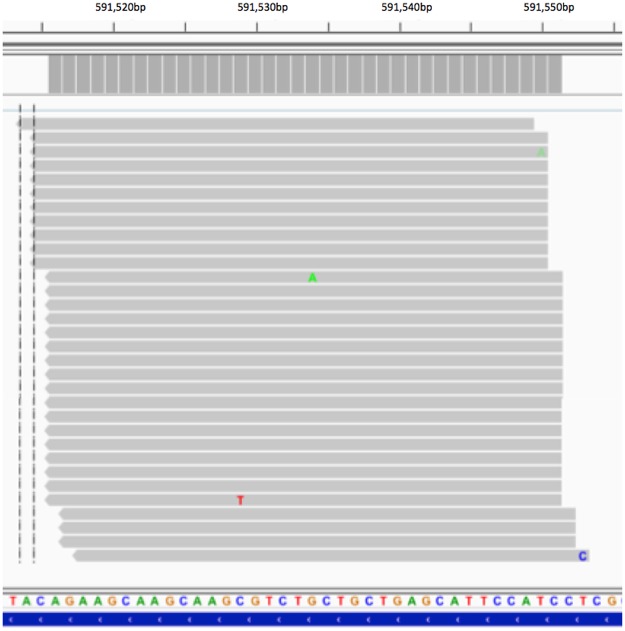
An example of reads sharing a UMI mapping to adjacent locations on the genome. The numbers of reads mapping to the five coordinates shown were 1, 10, 796, 3, and 1, respectively. The 796 alignments at the mode were edited to make it possible to see reads at each position in one figure. All reads in this UMI-read cluster were tagged with UMI AAAGGAGTGG. The central mapping location is Chr03:591516. The vertical dashed line was inserted by the application and indicates the coordinate used to search.

Processing of the read data in order to arrive at both read and UMI counts mapping to each *Physcomitrella patens* gene involved examining the genomic position of each read, and genomic position of each UMI. Since sequencing and PCR errors are known to be introduced into the UMI as well as into reads generally, we employed a method similar to that of Smith *et al*.^10^, in which UMI differing from one another by less than or equal to 1 bp and mapping to the same chromosomal location were considered to be the same UMI, differing due to mutation during PCR or by sequencing error. In the course of this process we observed that not only did reads having the same or closely-related UMI map to the same position as is to be expected, but they were also observed to map to multiple adjacent coordinates. Having one UMI map to distant coordinates is to be expected–UMI are expected to pair randomly with mRNA molecules, and there is a finite probability that multiple mRNA molecules will pair with the same UMI. However, having many instances of the same transcript starting within a few base pairs of one another and all choosing the same UMI appears much less likely. An example of this phenomenon from data set run_171108 is shown in Fig. 1, in which reads aligning to five positions on chromosome 3 all have the same UMI. This figure was made using the Integrative Genomics Viewer (IGV)^12^. Most of the reads mapping to the central location (Chr03:591516) were edited out to show reads at each position. The distribution of reads in this UMI-read cluster is curious; 796 reads map to the central location, with smaller numbers on either side, forming a bell-like curve. Our definition of a UMI-read cluster is a group of reads sharing a UMI mapping to a series of closely spaced coordinates, such that the majority of reads map to one location, with smaller numbers mapping closely upstream and downstream, forming a bell-shaped distribution. Based on this definition, the cluster in Fig. 1 is of size 5 (contains five adjacent mapping positions), has mapping shifts of strictly 1 (all spacings between adjacent mapping positions are 1 bp) and contains 811 reads.

**Figure 2:**
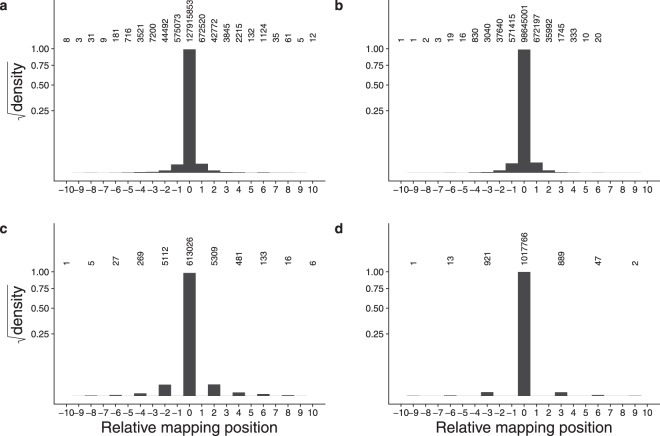
Mapping shifts of reads sharing a UMI in data set run_171108. (**a**) All UMI, including those having no mapping shifts. (**b**) UMI having adjacent mapping shifts of strictly 1 bp. (**c**) UMI having adjacent mapping shifts of strictly 2 bp. (**d**) UMI having adjacent mapping shifts of strictly 3 bp. The Y axis shows the square root of the probability density (summing to 1 for each plot), to make smaller values more visible. The position at which most of the reads map is position zero, with upstream mapping positions taking negative values, and downstream mapping positions taking positive values. Numbers of reads are indicated above each bar. The largest read cluster in this data set consisted of nine adjacent positions.

To investigate this further we examined all read mapping positions in the *Physcomitrella patens* genome and grouped reads into UMI-read clusters if they shared a UMI and mapped to genome coordinates within 3 bp of one another. Increasing the threshold above 3 has little effect on the distribution of the data. Supplementary Mapping Shift Frequencies file explains why this is so. It shows the distribution of spacings between read mapping coordinates sharing the same UMI. It can be seen that in our *P. patens* data sets run_171108 and run_170420 the great majority of shifts are of size 1, 2, and 3 bp, with the numbers rapidly decreasing with sizes beyond that. Shifts of larger size are present but rare and so have little impact on the distribution. In the other data sets the trend is similar, although not as dramatic. To make the analysis consistent across data sets we chose the 3 bp threshold. This threshold is a configurable parameter in our UMI processing pipeline, so the effect of clustering with various thresholds can be explored freely. The data for all UMI-read clusters were pooled by summing the number of reads mapping to the modal positions and to each position up- and downstream of the mode. Each modal mapping coordinate was normalized to zero, with coordinates to the left taking negative integers and positions to the right taking positive integers. This enabled superimposition of all clusters with respect to one another. Read numbers were converted to proportions of the total to normalize the sum to 1. The distribution is shown in Fig. 2a. The majority of reads map to the modal position, with diminishing numbers up- and downstream with increasing distance. A UMI-read cluster can be of size 1 (having no such shifts) meaning all reads for that UMI map to the same position. Shifts between adjacent positions may also differ in size. Figure 2b,c,d show the distributions of mapping shifts where the distances between adjacent mapping positions are strictly 1, 2, or 3 bp, respectively. Clusters having mapping shifts of strictly *n* are those in which all spacings between adjacent mapping coordinates are equal to *n*. For example, the UMI-read cluster illustrated in Fig. 1 has shifts of strictly 1. Mapping shifts of strictly 1, 2, or 3 were determined to explore the hypothesis that shifts of uniform intervals may reflect an underlying mechanism based on repeat-stabilized PCR stutter caused by repeats of different units.

### Read mapping shifts occur with multiple RNA-Seq protocols

To explore whether this phenomenon is seen in other RNA-Seq data sets using UMI, we analysed our other *P. patens* data (run_170420), three mouse data sets and one *C. elegans* data set obtained from the Sequence Read Archive (SRA). We chose two of these that were used by Smith *et al*. in the development of UMI-tools^10^. The first mouse data set consisted of 50 mouse midbrain SRA accessions from BioProject PRJNA307121^13^ (the La Manno data set). This data set was sequenced with the tagmentation protocol from which we derived ours^3^. The second data set is a single SRA accession (SRR1058003) from BioProject PRJNA232531, which was sequenced with the SCRB-seq protocol^14^ (the SCRB data set). A third data set consisted of 48 mouse SRA accessions from BioBroject PRJNA313513 from the laboratory of Yanai, who developed the CEL-Seq2 protocol for single cell transcriptomics^5^. The final *C. elegans* data set is also from the laboratory of Yanai. The SRA RNA-Seq data were aligned to the mouse and *C. elegans* genomes and the data processed similarly to our own. A summary of statistics of each data set is shown in Table 1. The data sampled span animals and plants, three species, small to large data sets, UMI from 6–16 bp, and three RNA-Seq protocols.

**Table 1:** Summary statistics of the data sets studied. Reads are numbers uniquely aligning (to one location in the genome).

Data Set	Species	Reads	Protocol	UMI bp	Read bp
run_171108	*P. patens*	158,663,380	Tagmentation	10	36
run_170420	*P. patens*	49,729,975	Tagmentation	10	36
SCRB	*M. musculus*	1,428,643	SCRB-seq	16	34
La Manno	*M. musculus*	3,332,377	Tagmentation	6	42
Yanai1	*M. musculus*	65,677,310	CEL-Seq2	6	35
Yanai2	*C. elegans*	1,445,609	CEL-Seq2	6	35

**Figure 3:**
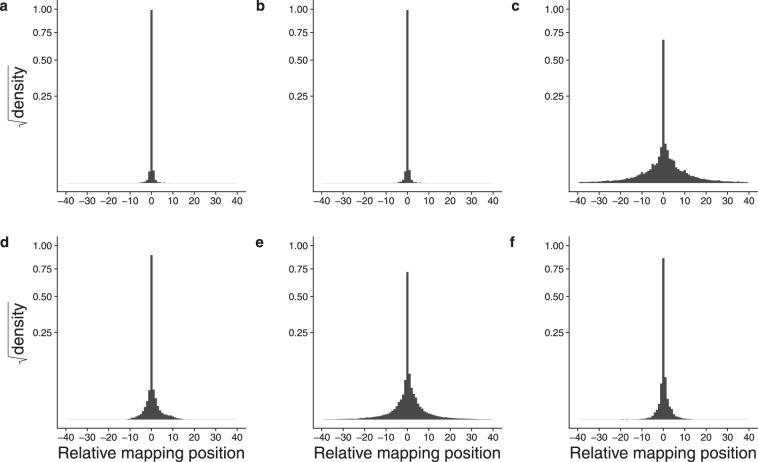
Mapping shifts of reads sharing a UMI in six data sets. Mapping shifts of all sizes are shown. The Y axis shows the square root of the probability density (summing to 1 for each plot), to make smaller values more visible. (**a**) run_171108. (**b**) run_170420. (**c**) SCRB. (**d**) La Manno. (**e**) Yanai1. (**f**) Yanai2. The numbers of reads in each category are shown in supplementary data file UMI Position Read Counts.

All of the data sets examined showed the mapping shift behaviour to varying extents. Fig. 3 shows the pooled data for the six sets. Data for all mapped reads were analysed to identify clusters sharing UMI but mapping to distinct adjacent coordinates. Data for each set were pooled and are shown as densities summing to one, to facilitate comparisons of data sets differing in size. The majority of reads map to the central location (no mapping shift) with significant numbers mapping to either side. Some data sets showed more variation than others, with standard deviations ranging from 0.12 (run_711108) bp to 3.7 (SCRB) bp. Thus, in all data sets observed, reads originating from the same starting mRNA molecule have related but different sequences.

**Figure 4:**
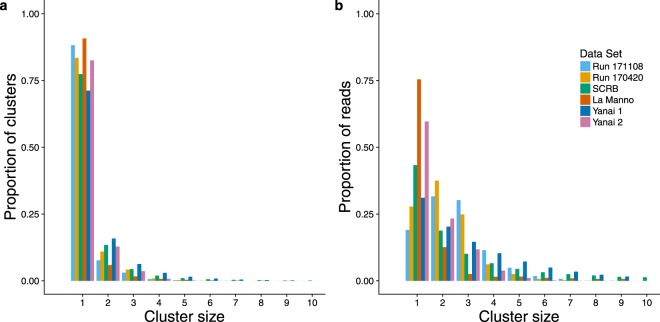
Proportions of clusters of different size and proportions of reads contained in clusters of different size. Cluster sizes range from 1, in which all reads having the same UMI map to the same coordinate, to a case in which reads having the same UMI map to a series 57 adjacent coordinates. The numbers of very large clusters are relatively small. Accordingly, cluster sizes up to 10 adjacent coordinates are shown here. (**a**) Proportions of clusters of different size. (**b**) Proportions of reads found in clusters of different size.

To further explore how reads are distributed among clusters of different sizes we plotted the number of clusters of each size, and the number of reads contained within each cluster size for each of the six data sets. The results are shown in Fig. 4. The numbers of clusters of each size is remarkably consistent across the data sets. However, the distribution of reads in each cluster size differs appreciably among them. For example, only 19% of reads in run_171108 are contained in clusters of size one (no mapping shift) with a majority of reads showing some mapping shift. In contrast, the La Manno data set has 75% of reads in clusters of size 1. The other data sets are intermediate.

### Read mapping shifts occur with multiple alignment algorithms

**Figure 5:**
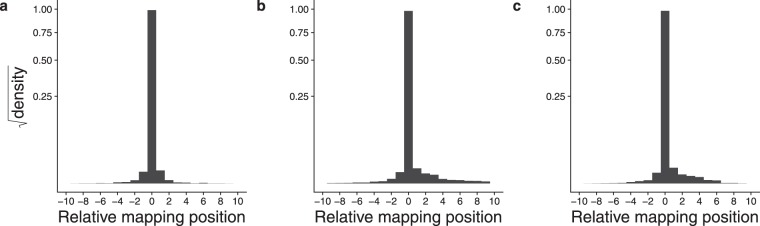
Mapping shifts of reads aligned to the genome with three algorithms. The Y axis shows the square root of the probability density (summing to 1 for each plot), to make smaller values more visible. (**a**) STAR. (**b**) GSNAP. (**c**) HISAT2. The numbers of reads in each category are shown in supplementary data file UMI Position Read Counts.

To determine whether the clustering behavior could be an artefact of a particular aligner, we aligned the run_170420 *P. patens* data set using STAR^15^, GSNAP^16^ and HISAT2^17^. Figure 5 shows that although the details differ as may be expected due to the different alignment characteristics of the three algorithms, the basic pattern is the same, with a pronounced mode and reads mapping to either side.

**Figure 6:**
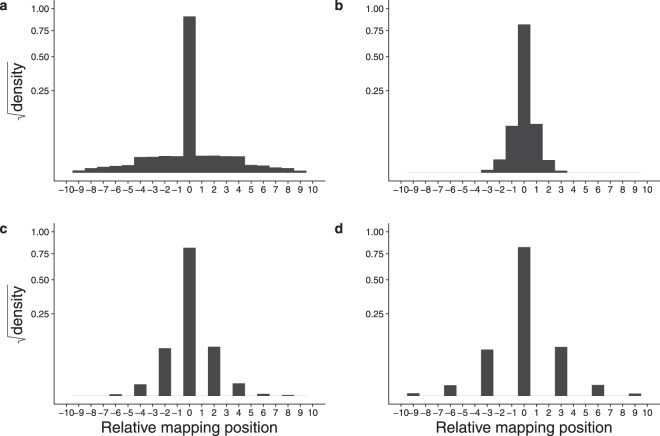
Mapping shifts of reads sharing a UMI in human plasmid data set SRR6704709 and SRR6704710. (**a**) All UMI, including those having no mapping shifts. (**b**) UMI having adjacent mapping shifts of strictly 1 bp. (**c**) UMI having adjacent mapping shifts of strictly 2 bp. (**d**) UMI having adjacent mapping shifts of strictly 3 bp. The Y axis shows the square root of the probability density (summing to 1 for each plot), to make smaller values more visible. The numbers of reads in each category are shown in supplementary data file UMI Position Read Counts.

Our interest is gene expression profiling with single cell precision; accordingly, we focused on our own RNA-Seq experiments and other RNA-Seq data in the public domain. However, UMI also offer utility in DNA sequencing applications, and we sought to explore the possibility that this same mapping shift phenomenon could occur with DNA-Seq. We obtained data for a single NCBI BioSample consisting of two SRA accessions, one containing paired end 300 bp Illumina data and the other containing an 8 bp UMI belonging to the sequence reads (SRR6704709 and SRR6704710). This data is sparsely annotated in the SRA, but is said to be derived from a plasmid pool of human origin and has to do with development of methods for genome editing. Although this is not strictly a genomic DNA template it is not from an RNA-Seq experiment. Accordingly, after trimming the data to 50 bp we processed the forward read with our pipeline by aligning to the human genome reference version 38. The results, which are very similar to the RNA-Seq results, are shown in Fig. 6.

### Read mapping shifts cause apparent over-expression of genes

**Figure 7:**
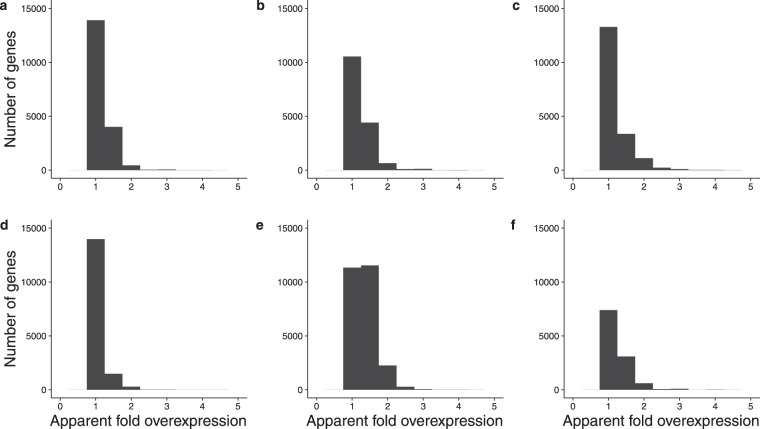
Apparent relative overexpression of genes if mapping shifts are not taken into account. UMI mapping to genes were counted with or without collapsing UMI-read clusters into single observation. The numbers of genes and their ratios of non-collapsed to collapsed UMI-read clusters are plotted. (**a**) run_171108. (**b**) run_170420. (**c**) SCRB. (**d**) La Manno. (**e**) Yanai1. (**f**) Yanai2.

To explore the impact on gene expression estimates of counting UMI mapping to multiple locations in the same gene as distinct events we counted UMI per gene with and without collapsing each UMI-read cluster into a single observation. We then treated the ratio of non-collapsed UMI counts to collapsed UMI counts as the relative expression level of each gene. The numbers of genes and their ratios of non-collapsed to collapsed UMI are plotted in Fig. 7. In all cases a considerable fraction of genes show artificially elevated gene expression levels. In the case of the Yanai1 data set the genes having apparent overexpression are in the majority. To evaluate the impact of this apparent overexpression we took six technical replicate *P. patens* RNA-Seq libraries and analysed them as two treatment groups, in which UMI-read clusters were collapsed into single observations or not. The collapsed and uncollapsed groups were analyzed with DESeq2^18^. The same analysis was done on 20 single cell data sets from the La Manno data. The results are shown in Supplementary Figs S3 and S4. The similarity matrices indicate that collapsed and uncollapsed treatments of the same sample are generally closer to one another than to other samples. The apparent overexpression in the uncollapsed data is evident in the MA plots; while the six-replicate *P. patens* data does not rise to the level of statistical significance, the twenty replicates from the La Manno mouse data contain 60 genes showing apparent overexpression with adjusted P values less than 0.05, strongly indicating that the same genes are reproducibly subject to the UMI-read clustering artefact in different samples.

To understand the impact of failing to collapse UMI-read clusters on differential gene expression analysis we studied two further RNA-Seq data sets obtained from the SRA. The first consisted of six replicates of mouse spleen cells treated with lipopolysaccharide (LPS) and six control replicates treated with phosphate buffered saline (PBS)^19^ (the Jaitin data set). The second data set consisted of three replicates of *in vitro* embryonic stem cells compared to three replicates of cells from the stromal fraction^20^ (the Nikaido data set). We analysed both data sets using DESeq2 to identify sets of genes differentially expressed at increasing levels of significance. This was done first with collapsing of UMI-read clusters into single observations, and a second time not correcting for this artefact. The results, shown in Supplementary Figs S5 and S6 show that at four chosen levels of significance, there are differences in the sets of genes identified as differentially expressed. In terms of the fraction of genes affected, the trend is more pronounced in the Jaitin data set, in which the difference makes up 17% of genes at *P* < 0.05 compared to 0.6% of genes in the Nikaido data set. The differentially expressed gene sets are shown in supplementary data files Nikaido Differential Expression and Jaitin Differential Expression. The gene identifiers therein refer to annotations in mouse assembly version GRCm38.p6.

### Read mapping shifts are associated with certain sequence characteristics

**Figure 8:**
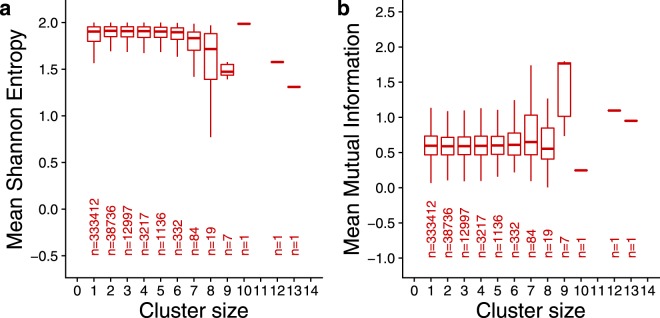
Shannon Entropy (**a**) and Mutual Information (**b**) of run_171108 reads belonging to clusters of increasing size. Each number on the X axis is the number of successive mapping coordinates in which reads share the same UMI. Cluster size = 1 represents reads mapping to only one location. Box plot hinges represent the first and third quartiles. Whiskers extend no more than 1.5 times the inter-quartile range.

All of the protocols considered here have in common that they utilize PCR to obtain sufficient material for sequencing. In another context, PCR is observed to result in a series of regularly-spaced products differing by a few base pairs–the presence of homopolymer runs and short tandem repeats is well known to cause repeat-stabilized mismatches resulting in PCR products that gain or lose length proportional to the repeat unit. This manifests itself as the familiar stutter seen in short tandem repeat markers (Fig. 1 in Bzymek and Lovett^21^ and Supplementary Fig. S2). Accordingly, we sought to determine whether reads in UMI libraries that demonstrate mapping shifts have sequence characteristics such as homopolymer runs and short tandem repeats. The Shannon Entropy of a character string measures the element of uncertainty with which a letter is expected to appear; strings containing reduced diversity have lower such uncertainty^22^. DNA sequences having lower than expected entropy include those containing homopolymers. For example, random 36-mers have average Shannon Entropy of 1.58, while perfect dinucleotide repeats have the value 1 and homopolymers have the value 0. Another useful quantity, also borrowed from information theory, is Mutual Information, which measures the influence of one distribution upon another^23^. By treating even and odd numbered bases as two random discrete variables, we can measure how strongly the identity of a nucleotide predicts the identity of the one following. Higher Mutual Information values are expected for reads in which the identity of a nucleotide strongly predicts the identity of the following nucleotide. For example, random 36-mers average Mutual Information of 0.29, while perfect dinucleotide repeats have the value 2.0. For reads forming clusters greater than size 1, we computed Shannon Entropy and Mutual Information for just the read at the mode. The results for run_171108 are shown in Fig. 8. As cluster size increases, there is a pronounced decrease in Shannon Entropy and an increase in Mutual Information. One-way analysis of variance (ANOVA) using the cluster size as the factor variable found that both results are highly significant (*P* < 10^−16^). ANOVA on the other data sets found similar results (Supplementary Figs S7 to S9). In the data sets where this trend is seen, it is more pronounced when shifts of strictly 2 bp are considered. Sequence reads found in large UMI-read clusters were extracted to visually inspect them for repeat content. Reads from each data set from clusters having shifts of strictly 2 bp are shown in Supplementary Tables S2 to S7. Different cluster size cut offs were chosen based on the size of each data set. There is obvious presence of dinucleotide repeats in the run_171108, run_170420, and Yanai1 data sets, while the reads in the La Manno data set are dominated by A and T homopolymer runs. Consistent with the absence of obvious trends in Shannon Entropy and Mutual Information in the SCRB and Yanai2 data sets (Supplementary Figs S7 and S8), the reads forming large clusters in those data sets lack obvious repeat content.

## Discussion

In the course of our single cell RNA-Seq studies in *Physcomitrella patens,* we discovered a phenomenon in which reads sharing a UMI, and therefore are derived from the same starting molecule, frequently map to a series of closely spaced mapping locations, forming bell-shaped distributions (Fig. 1). In this study, UMI made the discovery of this mapping shift phenomenon possible–in their absence, reads mapping to different coordinates are generally considered to be distinct from one another. A fundamental assumption in RNA-Seq has been that sequence reads having different mapping coordinates are derived from different starting molecules. Here we demonstrate that this assumption does not hold. The advantages of UMI in RNA-Seq regarding the elimination of PCR bias are well known^3,5^. Here we show that UMI reveal a further source of error with significant implications for read counting-based measures of gene expression. The benefits of UMI for eliminating PCR amplification bias are realized by collapsing reads originating from the same mRNA molecule into a single observation that counts an instance of that transcript. In order to do this, the originating mRNA is generally identified based on its starting mapping coordinate on the genome, based on the assumption that the sequencing read marks the 5′ end of a particular mRNA molecule. Identical UMI associated with reads having the same mapping coordinate are counted as one instance of expression of that gene. Some RNA-Seq software tools attempt to eliminate PCR duplicates and count reads making the assumption that reads having different mapping coordinates are derived from different starting molecules^3,5,10,24,25^. Using such reasoning, read mapping coordinates differing by a few bases will be counted as different gene expression instances, even if they share a UMI. We show here that the size of UMI-read clusters (the number of adjacent mapping coordinates of reads sharing a UMI) can be as large as 57, meaning that such a counting method will overestimate the expression level of some genes by a large factor.

The mapping shift phenomenon was observed in all RNA-Seq data sets that we studied, spanning plants and animals and three RNA-Seq protocols. All data sets displayed the mapping shift phenomenon, and to different extents. The SCRB-Seq data have the greatest spread, followed by the CEL-Seq2 data set Yanai1, the tagmentation data of La Manno, and the CEL-Seq2 data set Yanai2. Our tagmentation data have the narrowest distributions of those sampled. Thus, there is no obvious relationship between the spread and the RNA-Seq protocol employed. Cluster sizes were observed to differ in each data set, but the relative numbers of clusters in each size range were remarkably consistent among data sets (Fig. 4), hinting at some common underlying process. The number or reads belonging to clusters of differing size was more variable, but in some data sets, the number of reads mapping to the modal position are a minority of the total. The mapping shift phenomenon was observed in one non-RNA Seq experiment utilizing a plasmid template, and was recapitulated with three alignment algorithms, indicating that the source of the artefact is not intrinsic to RNA templates, nor is it an artefact of the alignment algorithm.

PCR products derived from the same template having lengths differing by multiples of 1–4 nucleotides is a well-known feature of templates containing simple tandem repeats^21^, and bears an uncanny similarity to our observation that UMI-read clusters can consist of several mapping positions differing by evenly-spaced gaps (Supplementary Fig. S2). Since all of the RNA-Seq library preparation protocols we studied use PCR to arrive at sufficient material to sequence, we explored the sequence composition of reads forming UMI-read clusters of increasing size with a view to identifying traits consistent with the presence of homopolymers and simple tandem repeats. As noted previously, random 36-mers have average Shannon Entropy of 1.58, while perfect dinucleotide repeats have the value 1 and homopolymers have the value 0. Random 36-mers average Mutual Information of 0.29, while perfect dinucleotide repeats have the value 2.0. We discovered a distinct tendency towards lower entropy as UMI-read clusters increased in size which is most pronounced in our run_171108 data and in the La Manno data set, but the data defy neat generalization, this tendency being less pronounced in the other data sets, despite highly significant P values derived from ANOVA. Mutual Information similarly was found to increase with increasing UMI-read cluster size in our data sets, and in the other data sets when only mapping shifts of 2 bp were analysed. The range of Shannon Entropy and Mutual Information values at each cluster size is large, so any conclusion about the contribution of PCR stutter caused by simple tandem repeats must be tempered by the caution that the mapping shifts can occur with reads of normal or high entropy, and with reads having low or normal Mutual Information. The role of PCR stutter generally could be further investigated using synthetic templates containing repeats of various kinds, and with libraries constructed with varying numbers of PCR cycles. Illumina sequencing itself uses bridge amplification to generate clusters on the flowcell, which could potentially be the step at which this artefact appears. However, it appears not to be dependent on the Illumina instrument or chemistry version. Illumina HiSeqs 2000 and 2500, and the MiSeq were used to generate data used in this study. The 2500 and MiSeq share a chemistry different from the earlier HiSeq 2000. The nature of the reads prone to forming clusters differs among the data sets studied. Examination of the reads forming larger clusters (greater numbers of adjacent mapping positions) showed presence of simple tandem repeat content, generally in keeping with the trends in Shannon Entropy and Mutual Information. However, the SCRB and Yanai2 reads were mostly devoid of such repeat content, despite forming clusters of significant size. Possibly, larger data sets are needed to observe meaningful trends in these metrics and in visible repeat content. Alternatively, repeat-stabilized PCR stutter may contribute to, but not fully explain the observed artefact.

The mechanism underlying formation of UMI-read clusters is deserving of further investigation but is of secondary importance. The main finding of this study is that by establishing a distinct identity for each starting mRNA, UMI revealed that a single starting template gives rise to reads that map to multiple adjacent locations in the genome. The widespread assumption that reads mapping to different sequence coordinates are derived from distinct mRNA molecules is incorrect. We grouped data into sets of six technical replicates (our run_171108 data) or twenty biological replicates (the La Manno data) and treated them as controls (collapsed UMI-read clusters) and cases (uncollapsed). Both the *P. patens* and mouse data sets show similar trends, in which a subset of genes shows apparent overexpression in the uncollapsed data due to over counting instances of the same transcripts. In the mouse data, this trend is statistically significant (*P* < 0.05) for 60 genes (Supplementary Table S8), which strongly suggests that the same subset of genes is subject to this artefact in these replicate samples. Quantitative gene expression analyses using UMI stand to benefit from elimination of PCR amplification bias, but our results indicate that reproducible overestimates of gene expression levels will occur unless this artefact is taken into account. Furthermore, in two treatment/control RNA-Seq data sets, comparing our method of collapsing UMI-read clusters into single observations to the standard practice showed that sets of genes identified as differentially expressed differed substantially at all levels of statistical significance studied. Thus, the artefact we have identified stands to affect such studies with both false positive and negative results. We conclude from these studies that UMI are not only valuable for eliminating PCR amplification bias, but also for increasing accuracy of gene sets identified as differentially expressed, but only if the appropriate corrections are made.

We are making available our analysis pipeline, which groups clusters of closely-spaced reads together as single observations. In our study, we considered reads to be derived from the same starting mRNA if they share a UMI and the mapping locations are separated by three or fewer base pairs. However, reads derived from the same template could have larger mapping shifts, the true distribution being unknown. Accordingly, we make this separation a configurable parameter. By adding this step to the process that harnesses the known advantages of UMI we hope to raise awareness of this artefact and to contribute to improved accuracy of quantitative gene expression studies.

## Methods

### RNA and cell Isolation

*Physcomitrella patens* tissue was cultured using standard methods according to Vidali *et al*.^26^. We used an NLS4 moss line expressing nuclear GFP for protoplasts fluorescence identification^27^. Total RNA was extracted via Quick-RNA MicroPrep (Zymoresearch) from 7 -day -old plant tissue. Plant tissue (about 300 mg) was frozen in liquid nitrogen, ground with a pre-chilled mortar and a pestle and homogenized in RNA lysis buffer. Single protoplasts were obtained as previously described^28^; briefly, plant tissue was digested with the enzyme cocktail, Driselease (0.5%) (Sigma), and dissolved in 8% mannitol. Using a spatula, 7 -day -old plant tissue was transferred into a petri dish containing 6 ml of Driselease solution; the petri dish was incubated at room temperature and gently shaken for 1 hour. The protoplast suspension was filtered through a 100 µm mesh (BD Falcon 352350). Protoplasts were washed in an 8% mannitol solution, with 3 consecutive centrifugation and resuspension of protoplast pellet in fresh mannitol. The washed protoplasts, resuspended in 8% mannitol, were pipetted into a holed microscope slide (Plexiglas) with a microscope coverslip (glass) attached at the bottom with vacuum grease. The microscope slide was placed onto an inverted epifluorescence microscope Axiovert 200 M (Zeiss). Protoplasts were extracted from the media with borosilicate glass capillaries (WPI, Inc), pulled with a flaming/brown micropipette puller, model-P-47 (Sutter Instrument). The glass capillary was connected to a manual pneumatic microinjector (CellTram Air Eppendorf). Single protoplasts were pipetted out of the mannitol suspension with the microinjector and pipetted into a PCR tube (VWR) containing 5 *μ*l of HBSS solution (Quality biological). The final volume was approximately 10 *μ*l. For detection of the protoplast in the tube, the cell was visualized by fluorescence GFP signal using a GFP filter cube GFP-30-LP-B-zhe zero (Semrock brightline). The tube was fast frozen in liquid nitrogen and stored at −80 °C until analysis.

### Tn5 enzyme purification

Tn5 transposase enzyme was extracted according to Picelli *et al*., 2014 with a few modifications^29^. Briefly, the pTXB1-Tn5 vector (Addgene) was transformed into T7 lysIq competent cells (NEB). Single colonies were picked and two litres of bacterial culture in LB was grown at 37 °C; when the cultures reached OD 0.9, cells were induced with IPTG 0.25 mM and grown at 24 °C until OD 2.5. Cells were collected and resuspended in 100 ml HEGX buffer (10 mM Tris-HCl pH 7.5, 700 mM NaCl, 1 mM EDTA, 10% glycerol, 1 mM DTT, 1 Roche tablet, 1 mM PMSF (Sigma), and DNAse) and lysed with Microfluidizer cell disruptor (Microfluidics) at 80 PSI. Cells were pelleted for 30 minutes at 34000 g. The supernatant was incubated with chitin resin (NEB) in a conical tube for 2 hr at 4 °C to favour binding (batch binding), then loaded on a column. The column was washed with 200 ml HEGX buffer, after which 12 ml of TGEX buffer with 100 mM DTT was loaded onto the column and 5.5 ml was drained out of the column. The column was left closed at 4 °C for 48 hr to ensure cleavage of the Tn5 from the intein. Elution was done in 1 ml aliquots and the concentration of the protein was measured via Bradford assay. The protein was concentrated with centrifugal filter units 10 K (Millipore). The activity of the protein was measured according to Picelli *et al*.,^29^.

### RNA preparation, reverse transcription and PCR

For run_170420 and run_171108, total RNA was extracted from *P. patens* as described above and diluted to 2.5 pg/*μ*l (1 cell equivalent) or 25 pg/*μ*l (10 cell equivalent) in RNase free water and 0.56 *μ*l of Takara RNase Inibitor (Takara cat. 2313 A) was added. The RNA was freeze-thawed three times at −80 °C.

For run_171108, individual protoplasts were harvested as described above in 5 *μ*l of Hank’s Balanced Salt Solution (HBSS) and 0.56 *μ*l of Takara RNase Inibitor was added. The protoplasts were lysed by freeze-thawing three times at −80 °C.

The following tagmentation protocol was derived from Islam *et al*.^3^, and was performed in sextuplicate using either purified *P. patens* RNA (technical replicates) or individual protoplast (biological replicates). For reverse transcription, 1 cell equivalent of RNA or protoplast lysate (~21 pg of RNA) or 10 cell equivalents (~210 pg of RNA) was mixed with template switching oligo, TSO3 (Supplementary Table S1) that contained 10 nucleotide UMIs. The mixtures were incubated in a thermocycler at 72 °C for three minutes and then slow ramped to 42 °C at 0.1 °C/second. Once the mixture reached 42 °C, the temperature was held for two minutes.

During the slow ramp incubation, an RT master mix (1X SuperScript II First Strand Buffer (Thermo Scientific 18064014), 3 mM MgCl_2_ (Thermo Scientific AB0359), 1.5 mM each of dNTP mix (Invitrogen 18427013), 4 mM DTT (Invitrogen 18064014), 18 U/*μ*l SuperScript II reverse transcriptase (Invitrogen 18064014), and 4 *μ*M FSP primer (Supplementary Table S1)) was made. After the RNA/TSO3 mixture was incubated for two minutes at 42 °C, the RT master mix was also placed on the thermocycler and incubated at 42 °C for an additional minute. Next, RT master mix was added to each of the six replicate RNA/TSO3 mixtures. The RT reactions were incubated at 42 °C for 90 minutes, followed by 70 °C for 10 minutes to inactivate the RT enzyme. The RT reactions were stored overnight at −20 °C.

The following day, half of the cDNA for each replicate was used for PCR (1X Advantage 2 PCR buffer (Takara 639201), dNTP mix 0.44 mM each (Invitrogen 18427013), 0.52 *μ*M MODIPCR primer (Supplementary Table S1), 2X Advantage 2 polymerase mix (Takara 639201)). The PCR reactions were placed in a thermocycler and run with the following cycling parameters: Step 1: 95 °C for two minutes, Step 2: 98 °C for 30 seconds, Step 3: 60 °C for 20 seconds, Step 4: 70 °C for four minutes, Step 5: Repeat Steps 2–4 for 28 cycles, Step 6: 70 °C for five minutes, and Step 7: hold at 4 °C. During Step 4, at cycles 21, 23, 25, 27, and 29, 5 *μ*l aliquots were collected and placed on ice. The aliquots were placed back on the thermocycler at Step 6 for a final extension step. The PCR aliquots for each replicate were run in a 1% TAE agarose gel. The gel was stained with ethidium bromide/H_2_O solution and imaged on a Bio-rad Gel Doc XR+ imaging system. From the gel images, 27 cylces was determined to be the optimal number of PCR cycles for purified RNA and protoplasts.

Following PCR optimization, the remaining cDNA for each replicate was used to repeat the PCR reactions described above. The cycling parameters were also repeated as described above but with 27 PCR cycles and without collecting aliquots. The PCR reactions were cleaned using 0.7X volumes of AmPure XP beads (Beckman Coulter A63881) and washed twice with 80% ethanol. The beads were incubated at room temperature for 7–15 minutes until they were completely dry. The PCR products were eluted in 14 *μ*l of EB buffer (Qiagen 19086). 12 *μ*l of eluted PCR products were collected.

1 *μ*l of PCR product for each replicate was quantified using a Qubit DNA High Sensitivity Assay Kit (ThermoFisher Scientific Q32851) and the Qubit3.0 fluorometer (ThermoFisher Scientific Q33216), as specified by the manufacturer. To assess the size distribution of the libraries, 1 *μ*l of each library was run on a 2100 Bioanalyzer (Agilent G2939A) using a High Sensitivity DNA Analysis kit (Agilent 5067–4626), as specified by the manufacturers’ instructions. The size distribution of the libraries ranged from 200–1000 bps. The libraries were stored overnight at −20 °C.

### Tagmentation and library amplification

The following day, the PCR product was simultaneously fragmented and barcoded using Tn5 DNA transposase to transfer Illumina adaptors to the target DNA. For each replicate, PCR product was mixed with tagmentation buffer (50 mM TAPS-NaOH pH 8.5 (Sigma T130-25G), 25 mM MgCl_2_ (Ambion Am9530G), and 40% PEG 8000 (Sigma 83271–500 ml-F)), and 1X Tn5 assembly (10X Tn5 assembly Stock- 6.25 *μ*M barcoded adaptor, dialysis buffer (100 mM HEPES-KOH, pH 7.2, 0.2 mM NaCl, 0.2 mM EDTA, 2 mM DTT, 20% glycerol), 40% glycerol, and 6.25 *μ*M Tn5 transposase). Each replicate received a Tn5 assembly containing a uniquely barcoded adapter. The reactions were incubated in a thermocycler at 55 °C for seven minutes and then transferred to ice. Proteinase K (0.5 *μ*g/*μ*l (Ambion AM2546)) was added to each reaction. The samples were incubated at 55 °C for seven minutes to inactivate the Tn5 transposase.

Dynabeads M-280 Streptavidin beads (Invitrogen 11206D) were washed once with BWT (10 mM Tris-HCl, pH 7.5 (Invitrogen 15567–027), 1 mM EDTA (Ambion AM9260G), 2 M NaCl (Ambion AM9760G), 0.02% Tween-20 (Sigma P9416-50 ML)) and were resuspended in BWT.

Washed Dynabeads M-280 Streptavidin beads were added to each tagmentation reaction and the mixture was rotated at room temperature for 10 minutes to allow the tagmentation libraries to bind to the beads. The bead slurries were placed on a DyaMag-96 side magnet for five minutes until the supernatant cleared. The supernatant was discarded and the beads were resuspended in PB buffer (Qiagen 19066). The bead slurry was placed back on a DyaMag-96 side magnet until the supernatant cleared. The supernatant was discarded and the beads were washed twice with TNT buffer (20 mM Tris-HCl, pH 7.5 (Invitrogen 15567–027), 50 mM NaCl (Ambion AM9760G), 0.02% Tween-20 (Sigma P9416-50 ML)).

The beads were resuspended in restriction mix (1X NEB Buffer 4 (New England Biolabs B7004S) and 0.4 U/*μ*l PvuI-HF (New England Biolabs R3150S)) to digest and remove unwanted 3′ fragments carrying the PvuI recognition site. The bead slurry was place in a thermocycler and incubated at 37 °C for one hour. After incubation, the bead slurry was placed on a DyaMag-96 side magnet until the supernatant cleared. The supernatant was discarded and the beads were washed three times with TNT, as described above. After the third TNT wash, the beads were resuspended in EB buffer.

Next, the tagmentation libraries were amplified by PCR to obtain enough material for Illumina sequencing. Each tagmentation library was mixed with 1X Advantage 2 PCR buffer, dNTP mix 0.2 mM each, 0.1 *μ*M P5 primer (Supplementary Table S1), 0.1 *μ*M P7 primer (Supplementary Table S1) and 1X Advantage 2 polymerase mix. The PCR reactions were placed in a thermocycler and run with the following cycling parameters: Step 1: 95 °C for two minutes, Step 2: 98 °C for 30 seconds, Step 3: 60 °C for 20 seconds, Step 4: 70 °C for four minutes, Step 5: Repeat Steps 2–4 for 9 cycles, Step 6: 70 °C for five minutes, and Step 7: hold at 4 °C overnight.

The following morning, the amplified libraries were cleaned with 0.7X volumes of AmPure XP beads and washed twice with 80% ethanol. The residual ethanol was removed and the beads were incubated at room temperature for 7–15 minutes until they were completely dry. The amplified libraries were eluted in 14 *μ*l of EB buffer and 12 *μ*l of library was collected.

The libraries were quantified using a Qubit DNA High Sensitivity Assay Kit and the Qubit3.0 fluorometer. The library size distribution was assessed using the 2100 Bioanalyzer and a High Sensitivity DNA Analysis kit. The size distribution of the libraries ranged from 200–700 bps. The libraries were stored at −20 °C until used for sequencing.

### Illumina Sequencing

Each library was quantified by 2100 Bioanalyzer, as described above. The libraries were normalized to 10 nM, pooled in equimolar amounts and denatured as described by Illumina’s “Denature and Dilute Libraries Guide” (Document 15050107 v03). The pools were clustered on a flowcell at 10 pM and sequenced on an Illumina HiSeq 2000 instrument using a TruSeq SBS Kitv3 (Illumina FC-401-3002, 50 cycles). MODR1P (Supplementary Table S1) was used as the read 1 primer. For run_171108, MODRIP (Supplementary Table S1) was used as the index read primer. 50 bp reads were generated for both runs along with 6 bp index reads for run_171108 corresponding to the RNA/protoplast-specific barcode.

### Data sets from the Sequence Read Archive

The La Manno data set (mouse, Illumina HiSeq 2000) is from BioProject PRJNA307121^13^, consisting of SRA accessions SRR4055118, SRR4055122, SRR4055126, SRR4055130, SRR4055134, SRR4055138, SRR4055142, SRR4055146, SRR4055150, SRR4055154, SRR4055158, SRR4055162, SRR4055167, SRR4055119, SRR4055123, SRR4055127, SRR4055131, SRR4055135, SRR4055139, SRR4055143, SRR4055147, SRR4055151, SRR4055155, SRR4055159, SRR4055164, SRR4055168, SRR4055120, SRR4055124, SRR4055128, SRR4055132, SRR4055136, SRR4055140, SRR4055144, SRR4055148, SRR4055152, SRR4055156, SRR4055160, SRR4055165, SRR4055121, SRR4055125, SRR4055129, SRR4055133, SRR4055137, SRR4055141, SRR4055145, SRR4055149, SRR4055153, SRR4055157, SRR4055161, SRR4055166. The SCRB data set (mouse, Illumina HiSeq 2000) is from BioProject PRJNA232531 consisting of SRA accession SRR1058003^14^. The Yanai1 data set (mouse, Illumina HiSeq 2500) is from BioBroject PRJNA313513^5^, consisting of SRA accessions SRR3196044, SRR3196045, SRR3196046, SRR3196047, SRR3196048, SRR3196049, SRR3196050, SRR3196051, SRR3196052, SRR3196053, SRR3196054, SRR3196055, SRR3196056, SRR3196057, SRR3196058, SRR3196059, SRR3196060, SRR3196061, SRR3196062, SRR3196063, SRR3196064, SRR3196065, SRR3196066, SRR3196067, SRR3196068, SRR3196069, SRR3196070, SRR3196071, SRR3196072, SRR3196073, SRR3196074, SRR3196075, SRR3196076, SRR3196077, SRR3196078, SRR3196079, SRR3196080, SRR3196081, SRR3196082, SRR3196083, SRR3196084, SRR3196085, SRR3196038, SRR3196039, SRR3196040, SRR3196041, SRR3196042, SRR3196043. The Yanai2 data set (*C. elegans*, Illumina HiSeq2500) is SRR3196113. The human plasmid data set combines SRR6704709 and SRR6704710 (Illumina MiSeq). The Jaitin data set consisted of six mouse spleen samples treated with LPS (SRR1106639, SRR1106638, SRR1106635, SRR1106634, SRR1106631, SRR1106630) and six controls (SRR1106629, SRR1106628, SRR1106637, SRR1106636, SRR1106633, SRR1106632). The Nikaido data set consisted of three embryonic stem cells (SRR6326639, SRR6326638, SRR6326637) and three stromal cells (SRR5664331, SRR5664330, SRR5664329).

### Computational pipeline

*Physcomitrella patens* version 3.3 genome and annotations were obtained from Phytozome (phytozome.jgi.doe.gov). NCBI provided the genomes and annotations of human (assembly GRCh38.p7), mouse (assembly GRCm38.p6), and *C. elegans* (assembly WBcel235). The following is a high level description of our analysis process that is further documented on GitHub and embodied in the code itself. Tagmentation data (our libraries and the La Manno data set) were processed as follows: reads having an average Phred score of less than 30 in the substring containing the UMI were discarded. The UMI is the most important part of the read in which to avoid error to ensure, as far as is possible, that reads sharing a UMI and mapping location are clonal in origin. The reads in this study were all short, and do not suffer from degradation of quality generally towards the end of the read. For fastq files in run_171708, run_170420, and La Manno data sets, the UMI was removed from the beginning of the read and placed in the fastq header. The subsequent 4 nucleotides containing the expected GGG(G) motif (our data) or 3 nucleotides (La Manno) were removed. The fastq quality string was truncated accordingly. For the SCRB, Yanai 1, Yanai 2 and Nikaido data sets, bespoke perl code was written to extract the UMI from one read and add it to the fastq header of the other. The Jaitin SRA files were extracted using the command fastq-dump–defline-seq ‘$ac.$si $sg’ SRRXXXXXX, which extracted the UMI into the header. The Nikaido reads were trimmed to 50 bp before alignment. Fastq files were aligned to the appropriate genomes with STAR (–runThreadN 16 –outFilterMismatchNmax 2 –alignIntronMax 1 –alignEndsType EndToEnd –outSAMattributes All), GSNAP (-N 1 -m 2 –format=sam), or HISAT2 (-x). Uniquely aligning reads were identified by retaining SAM lines matching pcregrep ‘\tNH:i:1\t’ file.sam, and non-uniquely aligning reads were discarded. Alignment data was then maintained in sorted SAM and BAM files. The sorted SAM file was parsed to identify reads sharing the same UMI mapping to the same coordinate in the genome. UMI mapping to the same coordinate were considered to be the same if the Levenshtein distance between them was no greater than 1. Genomic positions and the UMI mapping to those positions were recorded. This output was transformed into a format indicating UMI and their mapping positions in the genome. This output was parsed to identify sets of mapping positions each separated by no more than 3 bp sharing the same UMI (the UMI-read clusters). These clusters were collapsed into a single observation of a UMI. BED format was used to record genomic coordinates and the number of de-redundified UMI mapping to each coordinate, start and stop coordinates being the same, and the number of UMI being recorded in a fourth field. Bedtools intersect was then used to identify the annotation feature “gene” in GFF files corresponding to the genomic mapping coordinates of the UMI. This provided UMI counts per gene tables that can be used for quantitative gene expression analysis. SAM data was parsed into BED format to record genomic coordinates and the number of uniquely-aligning reads mapping to each one. Bedtools intersect was further used to identify the annotation feature “gene” for coordinates having mapped reads. This provided the read counts per gene tables useful for quantitative gene expression analysis. Shannon Entropy of a sequence was calculated as:

1 $$H=\sum _{x\in X}\,p(x)lo{g}_{2}p(x)$$

where *X* = {*G*, *A*, *T*, *C*} and *p*(*x*) is the fraction of the nucleotide string consisting of nucleotide *x*. Odd-numbered strings were made even by eliminating the first character, then dividing the string into 2-mers {*x*, *y*} where *x*, *y* ∈ {*G*, *A*, *T*, *C*}. Mutual information was then computed as:

2 $$I(X,Y)=\sum _{x,y}\,p(x,y)lo{g}_{2}(p(x,y)/(p(x)p(y)))$$

where *p*(*x*, *y*) is the fraction of 2-mers in the string consisting of the nucleotides *x* followed by *y*, and *p*(*x*) and *p*(*y*) are the fractions of nucleotides *x* and *y* in the string. *X* and *Y* are the odd and even numbered nucleotides in a sequence treated as two discrete random variables. Perl code was written to automate exploration of the findings reported. R was used for statistical analysis of the data and for the generation of graphics. Automated generation of R scripts was done with perl code. R scripts were executed with Rscript at the command line to produce text and image outputs.

## Electronic supplementary material

Supplementary Information

Mapping Shift Frequencies

UMI Position Read Counts

Jaitin Differential Expression

Nikaido Differential Expression

## Data Availability

Fastq files containing UMI in the headers, for run_171108 and run_170420 were deposited in SRA, study SRP150352. Individual accession numbers are SRR7295917, SRR7295918, SRR7295919, SRR7295920, SRR7295921, SRR7295922, SRR7295923, SRR7295924, SRR7295925, SRR7295926, SRR7295931, SRR7295932, SRR7295933, SRR7295934, SRR7295927, SRR7295928, SRR7295929, SRR7295930, SRR7295935, SRR7295936, SRR7295908, SRR7295907, SRR7295910, SRR7295909, SRR7295912, SRR7295911, SRR7295914, SRR7295913, SRR7295916, SRR7295915, SRR7295939, SRR7295940, SRR7295937, SRR7295938, SRR7295905, SRR7295906, SRR7295941. Further details are in Supplementary Table S9. The computational pipeline code is available on GitHub (https://github.com/ncgr/UMI-analysis).
