# Supplementary material for: Unique Molecular Identifiers reveal a novel sequencing artefact with implications for RNA-Seq based gene expression analysis
Source: Sci Rep. 2018 Sep 3;8:13121. doi: 10.1038/s41598-018-31064-7 (PMC6120941; doi:10.1038/s41598-018-31064-7)
Supplement: Supplementary file 1 — Supplementary Information [file 41598_2018_31064_MOESM1_ESM.pdf]

Unique Molecular Identifiers reveal a novel sequencing artefact with implications for RNA-seq based gene expression analysis

Johnny A. Sena[1], Giulia Galotto[2], Nico P. Devitt[1], Melanie C. Connick[1], Jennifer L. Jacobi[1], Pooja E. Umale[1], Luis Vidali[2], and Callum J. Bell[1,*]

[1]National Center for Genome Resources, Santa Fe, NM 87505, United States
[2]Worcester Polytechnic Institute, Department of Biology and Biotechnology, Worcester, MA 01609, United States
[*]cjb@ncgr.org

Supplementary Methods

Detailed Methods: Tn5 complex assembly and activity Assay

The TAG1 tagmentation oligo was order from IDT with standard desalting and 5' phosphorylation along with six barcoded tagmentation oligos,TAG2, that were ordered with standard desalting and analytical RP-HPLC (Supplementary Table S 1). Each tagmentation oligo was resuspended at 100 μM in Nuclease-free Duplex Buffer (IDT 11-05-01-12). Each of the six barcoded TAG2 oligos were mixed with equimolar amounts of TAG1 and incubated at 94°C in a thermocycler for two minutes to denature the oligos. The six oligo mixtures were removed from the thermocycler and placed on the benchtop for 30 minutes to anneal the TAG1 and TAG2 oligos and to form six uniquely barcoded hetero-duplexes. For each of the six hetero-duplexes, 11.94 μl of duplex (6.26 μM) was mixed with 24 μl of dialysis buffer, 80 μl of glycerol (42%), 72 μl of Tn5 transposase (0.7 μg/μl) and 3.06 μl of nuclease-free H_2O to make six uniquely barcoded transpososome assemblies. The transpososome assemblies were stored at -20 °C until tested for transposase activity.

To test the transposomes for transposase activity, the pDONR-222 (100 ng/μl) (Thermo Fisher Scientific -CloneMiner™ II cDNA Library Construction Kit A11180) or pUC19 (100 ng/μl) (Thermo Fisher Scientific SD0061) plasmid was linearized by PstI-HF (40 units) restriction digestion (New England Biolabs R3140S). The digested plasmid was loaded in a 1% TAE agarose gel and run for 45 minutes, stained with a 0.4 ng/μl ethidium bromide/H_2O solution for 20 minutes and de-stained with distilled H_2O for 15 minutes. The gel was imaged on a Bio-rad Gel Doc XR+ imaging system with Image Lab Software v.5.2.1. to confirm plasmid linearization. Next 100 ng of plasmid was mixed with 2 μl of each uniquely barcoded transposome assembly, 4 μl of tagmentation buffer (50mM TAPS-NaOH pH 8.5 (Sigma T130-25G), 25mM $MgCl_2$ (Ambion Am9530G), and 40% PEG 8000 (Sigma 83271-500ml-F)), and 13 μl of H_2O. The mixtures were incubated in a thermocycler at 55 °C for seven minutes and then place on ice. 0.5 μl of proteinase K (0.49 μg/μl) (Ambion AM2546) was added to the plasmid/tagmentation mixtures and incubated at 55 °C for seven minutes to inactivate the transposomes. The tagmented plasmids were run in 1% TAE agarose gel as described above to assess tagmentation efficiency. The transposomes displayed 100 % efficiency by completely fragmenting the linearized plasmid as indicated by a smear in the agarose gel. Functional transposomes were then used for Illumina library preparation as indicated below.

Detailed Methods: RNA preparation, reverse transcription and PCR

For run_170420 and run_171108, total RNA was extracted from *P. patens* as described above and serially diluted to 25 pg/μl for 1 cell equivalents and 250 pg/μl for 10 cell equivalents. 1 ul of the diluted RNA was resuspended in 9 μl RNase free water and 0.56 μl of Takara RNase Inibitor (Takara cat. 2313A) was added. The RNA was freeze-thawed three times at -80 °C and room temperature,respectively, to mimic the lysis conditions used for single protoplasts. Six technical replicates were processed at a time, three 1 cell equivalents and three 10 cell equivalents.

For run_171108, six individual protoplasts were harvested as described above in 5 μl of Hank's Balanced Salt Solution (HBSS) and 0.56 μl of Takara RNase Inibitor was added. The protoplasts were lysed by freezing and thawing three times at -80 °C and room temperature, respectively. Following the freeze-thaw cycles, the RNA and protoplasts were placed on ice.

For RT, 8.2 μl of RNA or protoplast lysate was mixed with 1.8 μl of 10 μM template switching RNA oligo , TSO3 (Supplementary Table S 1), in 200 μl PCR tubes. The mixtures were incubated in a thermocycler at 72°C with a heated lid for three minutes and then slow ramped to 42°C at 0.1°C/second. Once the mixture reached 42°C, the temperature was held for two minutes.

During the slow ramp incubation, an RT master mix was set up in a 200 μl PCR tube with the following reagents, 28.8 μl of 5X SuperScript II First Strand Buffer (Thermo Scientific 18064014), 17.28 μl of 25 mM $MgCl_2$ (Thermo Scientific AB0359), 21.6 μl of dNTP mix 10 mM each (Invitrogen 18427013), 5.76 μl of 0.1 M DTT (Invitrogen 18064014), 12.96 μl

of 200 Uμl SuperScript II reverse transcriptase (Invitrogen 18064014), and 5.76 μl of 100 μM FSP primer (Supplementary Table S 1). After the RNA/TSO3 mixture was incubated for two minutes at 42°C, the RT master mix was also placed on the thermocycler and incubated at 42°C for an additional minute. Next, 10 μl of RT master mix was added to each of the replicate RNA/TSO3 mixtures and mixed by pipetting. The RT reactions were incubated at 42°C for 90 minutes. Then, the RT reactions were incubated at 70°C for 10 minutes to inactivate the RT enzyme. The RT reactions were stored overnight at -20°C.

The following day, RT reactions were thawed on ice and used for PCR optimization. For each replicate, a 10 μl aliquot of cDNA was mixed with 2.5 μl of 10X Advantage 2 PCR buffer (Takara 639201), 1.1 μl dNTP mix 10 mM each (Invitrogen 18427013), 1.3 μl of 10 μM MODIPCR primer (Supplementary Table S 1), 9.1 μl of nuclease free H_2O, and 1 μl of 50X Advantage 2 polymerase mix (Takara 639201). The PCR reactions were placed in a thermocycler with a heated lid and run with the following cycling parameters: Step 1: 95°C for two minutes, Step 2: 98°C for 30 seconds, Step 3: 60°C for 20 seconds, Step 4: 70°C for four minutes, Step 5: Repeat Steps 2-4 for 28 cycles, Step 6: 70°C for five minutes, and Step 7: hold at 4°C. During Step 4, at cycles 21, 23, 25, 27, and 29, 5 μl aliquots were collected and placed on ice. The aliquots were placed back on the thermocycler at Step 6 for a final extension step. The PCR aliquots for each replicate were run in a 1% TAE agarose gel, stained with a 0.4 ng/μl ethidium bromide/H_2O solution for 20 minutes and de-stained with distilled H_2O for 15 minutes. The gel was imaged on a Bio-rad Gel Doc XR+ imaging system with Image Lab Software v.5.2.1. From the gel images, 27 cylces was determined to be the optimal number of PCR cycles for purified RNA and protoplasts.

Following PCR optimization, the remaining cDNA for each replicate, 10 μl each, was used to repeat the PCR reactions described above. The cycling parameters were also repeated as described above but with 27 PCR cycles and without collecting aliquots. The PCR reactions were cleaned by adding 17.5 μl of AmPure XP beads (Beckman Coulter A63881) to each 25 μl PCR reaction. The PCR/bead slurry was mixed until homogeneous by pipetting or gentle flicking of the PCR tubes. The slurry tubes were centrifuged briefly to collect droplets and were incubated at room temperate for five minutes. Following incubation, the PCR tubes containing the slurry were placed on a DyaMag-96 side magnet (ThermoFisher Scientific 12331D) for five minutes until the supernatant was clear. While on the magnet, the cleared supernatant was discarded, 200 μl of 80% ethanol was added to the beads, and incubated on the magnet for 30 seconds. The ethanol was discarded and the beads were washed a second time with 80% ethanol, as described above. The ethanol was discarded and the PCR tubes were centrifuged briefly to collect residual ethanol. The tubes were placed back on the magnet and the residual ethanol was removed by pipetting. The tubes were left on the magnet for 7-15 minutes with the caps open until the beads were completely dry. The tubes were removed from the magnet. The beads were resuspended in 14 μl of EB buffer (Qiagen 19086) and mixed until homogeneous. The EB/bead slurries were incubated at room temperature for five minutes to elute the PCR product and then placed back on the magnet for two minutes until the supernatant was clear. 12 μl of cleared supernatant was transferred to new 200 μl PCR tubes.

1 μl of PCR product for each replicate was quantified, as specified by the manufacturer, using a Qubit DNA High Sensitivity Assay Kit (ThermoFisher Scientific Q32851) and the Qubit3.0 fluorometer (ThermoFisher Scientific Q33216). To assess the size distribution of the libraries , 1 μl of each library was run on a 2100 Bioanalyzer (Agilent G2939A) using a High Sensitivity DNA Analysis kit (Agilent 5067-4626) as specified by the manufacturers' instructions. The size distribution of the libraries ranged from 200-1000 bps. The libraries were stored overnight at -20°C.

Detailed Methods: Tagmentation and library amplification.

The following day, the PCR product was thawed on ice. The PCR product was simultaneously fragmented and barcoded using Tn5 DNA transposase to transfer adaptors to the target DNA. For each replicate, 10 μl of PCR product was mixed with 4 μl of tagmentation buffer (50mM TAPS-NaOH pH 8.5 (Sigma T130-25G), 25mM $MgCl_2$ (Ambion Am9530G), and 40% PEG 8000 (Sigma 83271-500ml-F) in nuclease free H_2O), 2 μl of 10X Tn5 assembly, with each replicate receiving an assembly containing a uniquely barcoded adapter, and 4 μl of nuclease free H_2O in 200 μl PCR tubes. The reactions were incubated in a thermocycler at 55°C for seven minutes and then transfered to ice. 0.5 μl of 20 mg/ml proteinase K (Ambion AM2546) was added to each reaction. The reactions were mixed by gently flicking the tubes and centrifuged briefly to collect droplets. The samples were placed back in a thermocycler and incubated at 55°C for seven minutes and then placed on ice.

For six replicates, 120 μl of Dynabeads M-280 Streptavidin beads (Invitrogen 11206D) were pipetted into a 1.5 ml LoBind Eppendorf tube and placed on a DyanMag-2 magnet (ThermoFisher Scientific 12321D) until the supernatant cleared. The supernatant was discarded by pipetting. The tube was removed from the magnet and 120 μl of BWT (10 mM Tris-HCl , pH 7.5 (Invitrogen 15567-027)), 1 mM EDTA (Ambion AM9260G), 2 M NaCl (Ambion AM9760G), 0.02% Tween-20 (Sigma P9416-50ML)) was added to the beads and mixed by pipetting until the mixture was homogeneous. The bead slurry was placed back on the magnet until the supernatant cleared and the supernatant was discarded. The beads were removed from the magnet and were resuspended in 120 of BWT.

20 μl of washed Dynabeads M-280 Streptavidin beads were added to each 20 μl tagmentation reaction. The bead slurries were mixed by gently flicking the PCR tubes and centrifuged briefly to collect droplets. Then, the bead slurries were rotated at room temperature for 10 minutes to allow the tagmentation libraries to bind to the beads. The bead slurries were centrifuged

briefly to collect droplets and placed on a DyaMag-96 side magnet for five minutes until the supernatant cleared. The supernatant was discarded and the PCR tubes were removed from the magnet. Then, the beads were resuspended in 100 μl of PB buffer (Qiagen 19066) and mixed by pipetting. The bead slurry was place on a DyaMag-96 side magnet until the supernatant cleared. The supernatant was discarded. The bead containing tubes were removed from the magnet and the beads were resuspended in 100 μl of TNT buffer (20 mM Tris-HCl , pH 7.5 (Invitrogen 15567-027)), 50 mM NaCl (Ambion AM9760G), 0.02% Tween-20 (Sigma P9416-50ML)) and mixed by pipetting. The bead slurry was place back on the magnet for five minutes until the supernatant cleared. The supernatant was discarded and the TNT wash was repeated again.

This time, the beads were resuspended in 100 μl of restriction mix (1X NEB Buffer 4 (New England Biolabs B7004S) and 0.4 U/μl PvuI-HF (New England Biolabs R3150S) in H_2O) and mixed by pipetting. The bead slurry was place in a thermocycler and incubated at 37°C for one hour to digest and remove unwanted 3$'$ fragments carrying the PvuI recognition site. After incubation, the bead slurry was placed on a DyaMag-96 side magnet until the supernatant cleared. The supernatant was discarded and the beads were washed three times with TNT as described above. After the third TNT wash, the beads were resuspended in 20 μl of EB buffer. The eluted tagmenation library was placed on ice.

Next, the tagmentation libraries were amplified by PCR to obtain enough library for Illumina sequencing. For each replicate, 20 μl of tagmentation library was mixed with 5 μl of 10X Advantage 2 PCR buffer (Takara 639201), 1 μl dNTP mix 10 mM each (Invitrogen 18427013), 0.5 μl of 10 μM P5 primer (Supplementary Table S 1), 0.5 μl of 10 μM P7 primer (Supplementary Table S 1), 22 μl of nuclease free H_2O, and 1 μl of 50X Advantage 2 polymerase mix (Takara 639201). The PCR reactions were placed in a thermocycler with a heated lid and run with the following cycling parameters: Step 1: 95°C for two minutes, Step 2: 98°C for 30 seconds, Step 3: 60°C for 20 seconds, Step 4: 70°C for four minutes, Step 5: Repeat Steps 2-4 for 9 cycles, Step 6: 70°C for five minutes, and Step 7: hold at 4°C overnight. The following morning, the amplified libraries were removed from the thermocycler and placed at room temperature.

The amplified libraries were cleaned by adding 35 μl of AmPure XP beads to each 50 μl PCR reaction. The PCR/bead slurry was mixed until homogeneous by pipetting or gentle flicking of the PCR tubes. The slurry tubes were centrifuged briefly to collect droplets and were incubated at room temperature for five minutes. The PCR/bead slurries were placed on a DyaMag-96 side magnet for five minutes until the supernatant was clear. While on the magnet, the cleared supernatant was discarded by pipetting. 200 μl of 80% ethanol was added to the beads and the beads were incubated for 30 seconds. The ethanol was discarded and the beads were washed a second time with 80% ethanol, as described above. The PCR tubes were centrifuged briefly to collect residual ethanol and placed back on the magnet. The residual ethanol was removed by pipetting. The tubes were left on the magnet for 7-15 minutes with the caps open until the beads were completely dry. The tubes were removed from the magnet and were resuspended in 14 μl of EB buffer and mixed until homogeneous. The EB/bead slurries were incubated at room temperature for five minutes to elute the libraries and then placed back on the magnet for two minutes until the supernatant was clear. 12 μl of cleared supernatant was transferred to new 200 μl PCR tubes.

1 μl of each library was quantified using a Qubit DNA High Sensitivity Assay Kit and the Qubit3.0 fluorometer, as specified by the manufacturer. To assess the size distribution of the libraries , 1 μl of each library was run on a 2100 Bioanalyzer using a High Sensitivity DNA Analysis kit (see manufacturers' instructions). The size distribution of the libraries ranged from 200-700 bps. The libraries were stored at -20°C until used for sequencing.

Oligo Name	Sequence
FSP	AAT GAT ACG GCG ACC ACC GAT CGT TTT TTT TTT TTT TTT TTT TTT TTT TTT TTT
P5	AAT GAT ACG GCG ACC ACC GA
P7	CAA GCA GAA GAC GGC ATA CGA GAT
TAG1	/5Phos/CTG TCT CTT ATA CAC ATC TGA CGC
TAG2-AD002	CAA GCA GAA GAC GGC ATA CGA GAT CGA TGT AGC GTC AGA TGT GTA TAA GAG ACA G
TAG2-AD004	CAA GCA GAA GAC GGC ATA CGA GAT TGA CCA AGC GTC AGA TGT GTA TAA GAG ACA G
TAG2-AD005	CAA GCA GAA GAC GGC ATA CGA GAT ACA GTG AGC GTC AGA TGT GTA TAA GAG ACA G
TAG2-AD006	CAA GCA GAA GAC GGC ATA CGA GAT GCC AAT AGC GTC AGA TGT GTA TAA GAG ACA G
TAG2-AD007	CAA GCA GAA GAC GGC ATA CGA GAT CAG ATC AGC GTC AGA TGT GTA TAA GAG ACA G
TAG2-AD012	CAA GCA GAA GAC GGC ATA CGA GAT CTT GTA AGC GTC AGA TGT GTA TAA GAG ACA G
MODRIP	CTG TCT CTT ATA CAC ATC TGA CGC T
MODR1P	GCG ACC ACC GAG ATC TAC AC
TSO3	rArArU rGrArU rArCrG rGrCrG rArCrC rArCrC rGrArG rArUrC rUrArC rArC(N1:25252525) (N1)(N1)(N1) (N1)(N1)(N1) (N1)(N1)(N1) rGrGrG

Table S 1. Reverse Transcription, Tagmentation, PCR and Template Switching Oligos.

Compute environment

Computations were done out on a server having 64 8-core CPUs (Intel Xeon E7-4830, 2.13GHz, 24 MB cache) with 1 GByte of RAM. The operating system was CentOS plus x86_64. Bioinformatics software was installed and managed in a Bioconda (bioconda.github.io) environment with the following packages: bamtools 2.4.0[1], bcftools 1.6 (http://www.sanger.ac.uk/science/tools/samtools-bcftools-htslib), bedtools 2.27.0[2], fastqc 0.11.5 (https://www.bioinformatics.babraham.ac.uk/projects/fastqc), fastx_toolkit 0.0.14 (http://hannonlab.cshl.edu/fastx_toolkit), gmap 2017.09.30[3], hisat2 2.1.0[4], htslib 1.5 (http://www.sanger.ac.uk/science/tools/samtools-bcftools-htslib), parallel 20170422[5], picard 2.14.1 (http://broadinstitute.github.io/picard/faq.html), samtools 1.5[6], sra-tools 2.8.2 (https://github.com/ncbi/sra-tools), star 2.5.3a[7], vcflib 1.0.0_rc1 (https://github.com/vcflib/vcflib), vcftools 0.1.14[8], Rscript 3.4.1[9], bioconductor-deseq2 1.18.1[10]. Our pipeline for fastqc manipulation, demultiplexing, quality filtering, alignment, and UMI analysis consisted of a series of shell scripts (interpreted with GNU bash version 4.1.2), Perl programs (interpreted with version 5.10.1), C programs (compiled using GCC 4.4.7) and R scripts (R 3.4.3 GUI 1.70 El Capitan build (7463)). R packages ggplot2[11] and dplyr[12] were used. This software is available and documented (including dependencies) on GitHub. DESeq2 analysis was done with modifications to a template provided by https://gist.github.com/stephenturner/f60c1934405c127f09a6#file-deseq2-analysis-template-r-L62

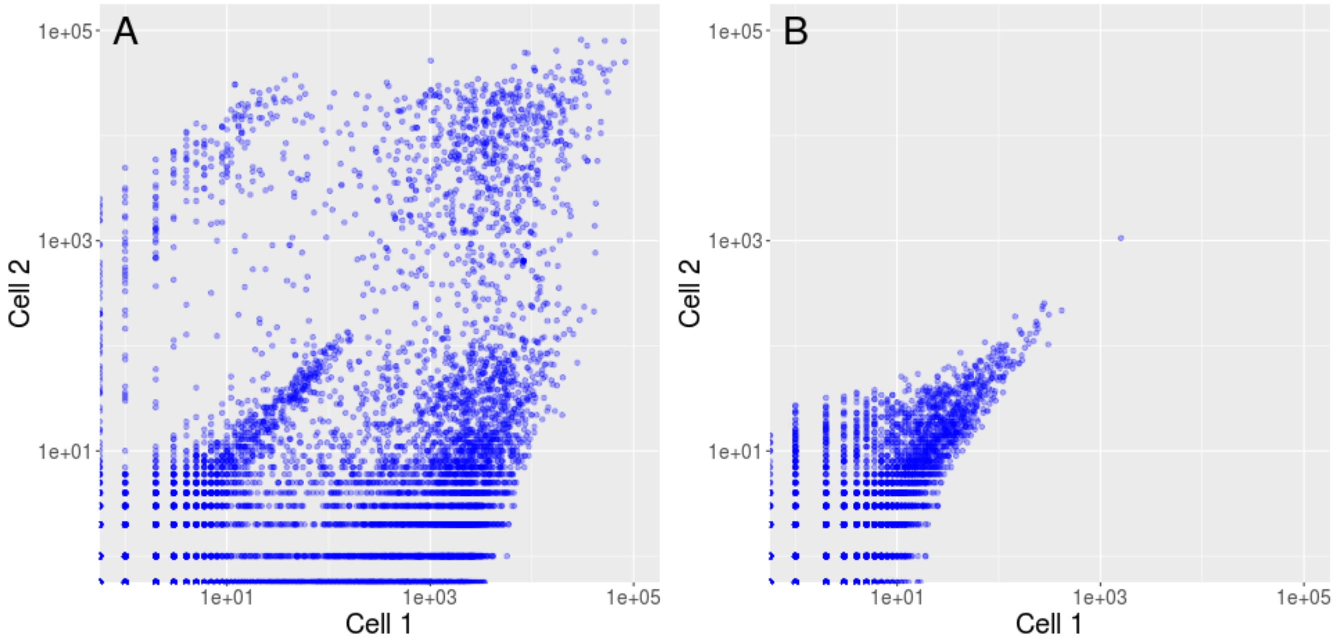

Figure S 1. Comparison of RNA-Seq UMI and read counts from sequencing data derived from two *P. patens* protoplasts. A: read counts per gene. B: UMI counts per gene. UMI and read counts are shown for the same data. Each dot plots a gene having two counts of reads or UMI as indicated by the X and Y axes.

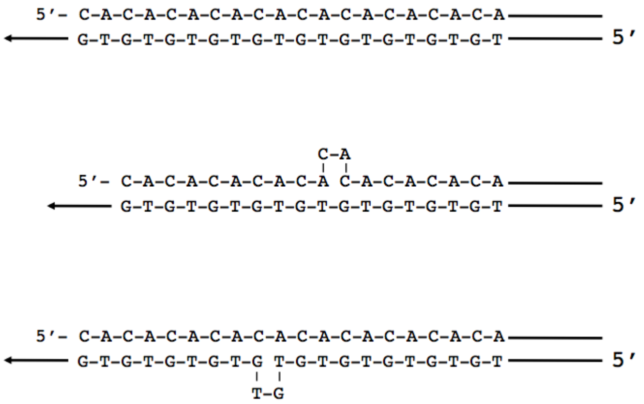

Figure S 2. Model of repeat-stabilized PCR stutter. Arrow indicates the direction of polymerization during a PCR cycle. The normal situation is indicated at the top, in which the template and copy strand are the same length. Slippage causing looping out of the template (center) or the copy strand (bottom) leads to products differing from the template by multiples of -2 and +2 respectively. An example of the repeat $(CA)_n$ is shown, although this may happen with any simple tandem repeat, including homopolymers.

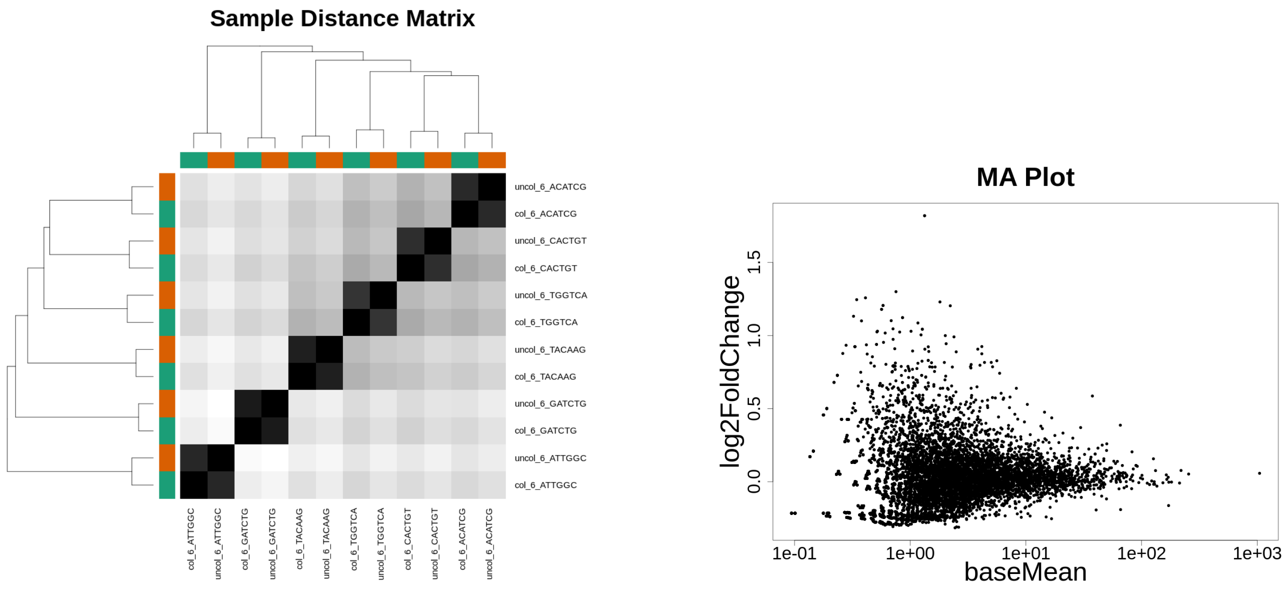

Figure S 3. DESeq2 analysis of six *P. patens* RNA-seq technical replicates from the run_171108 data set in which UMI-read clusters were collapsed (col_) or not collapsed (uncol_) into single observations. Left: pairwise distance matrix. Right: MA plot with log_2 fold *apparent* expression differences on the Y axis and mean expression level on the X.

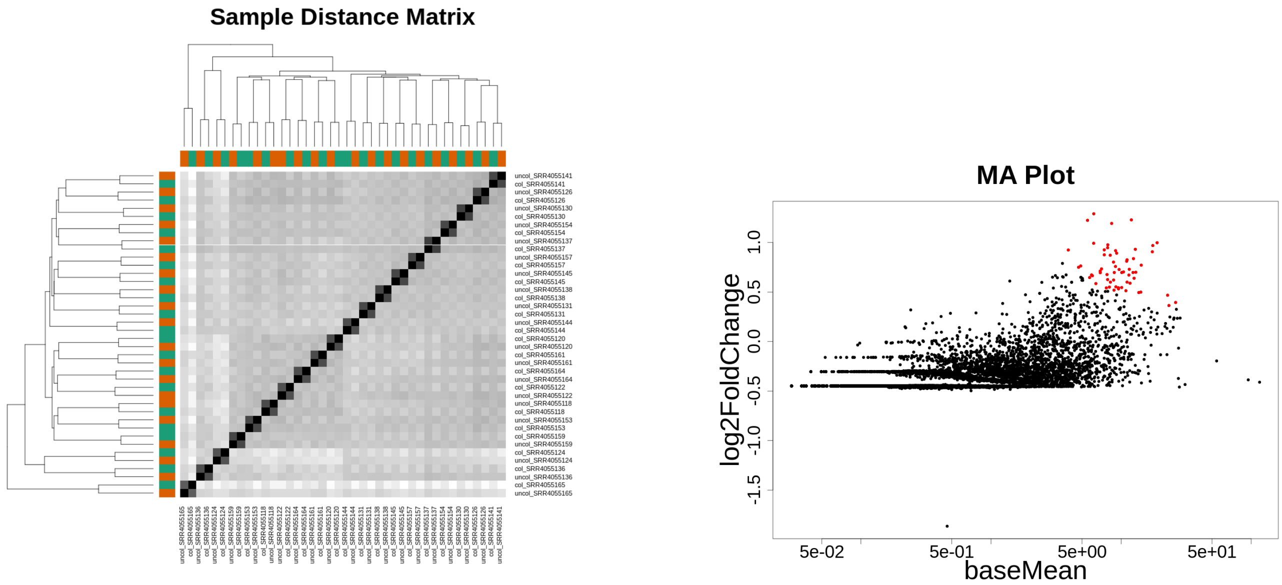

Figure S 4. DESeq2 analysis of 20 mouse RNA-seq biological replicates (single cells) from the La Manno data set in which UMI-read clusters were collapsed (col_) or not collapsed (uncol_) into single observations. Left: pairwise distance matrix. Right: MA plot with log_2 fold *apparent* expression differences on the Y axis and mean expression level on the X. Genes having adjusted P values of less than 0.05 have red dots.

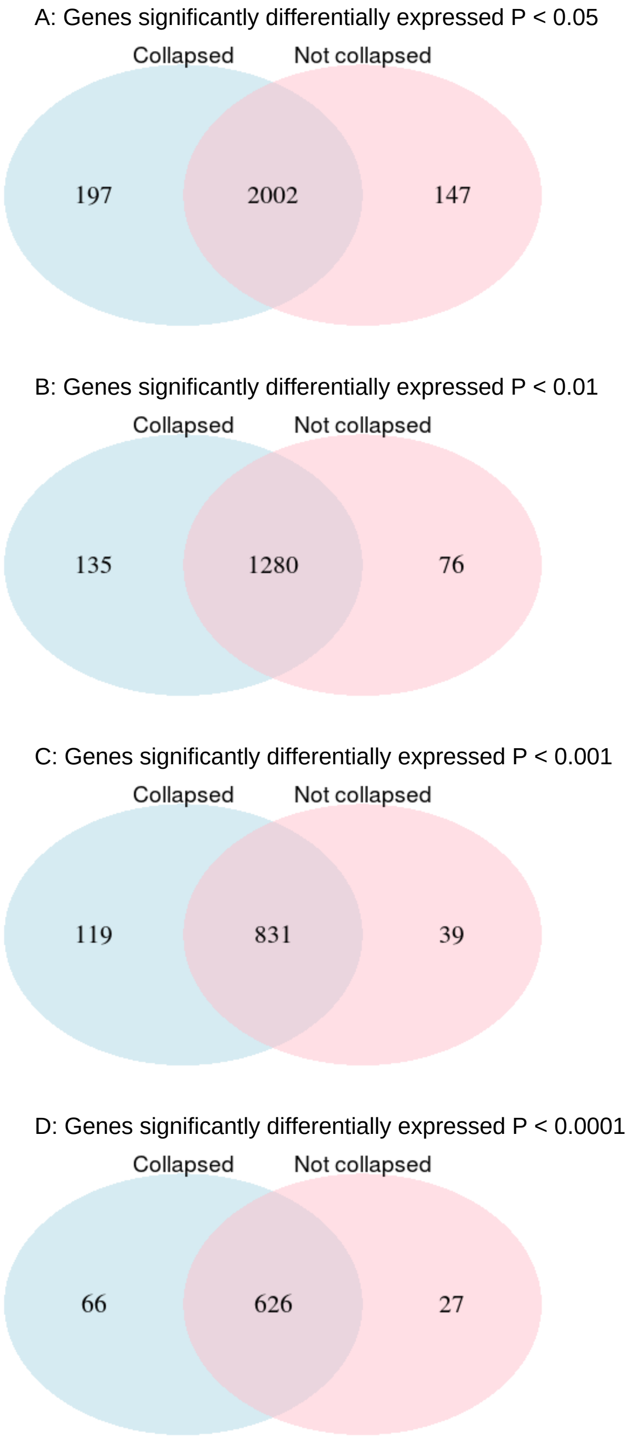

Figure S 5. Intersections and differences between sets of genes differentially expressed at four levels of statistical significance in the Jaitin data set when UMI-gene clusters are collapsed or not collapsed into single observations.

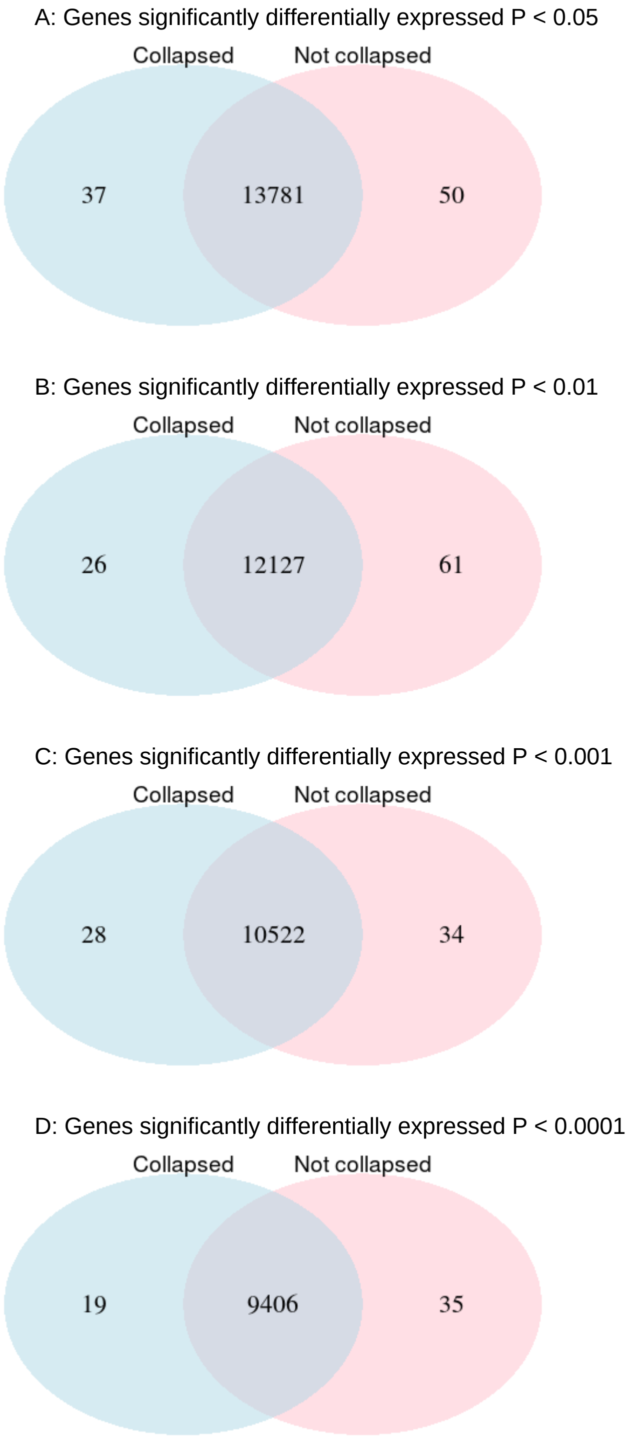

Figure S 6. Intersections and differences between sets of genes differentially expressed at four levels of statistical significance in the Nikaido data set when UMI-gene clusters are collapsed or not collapsed into single observations.

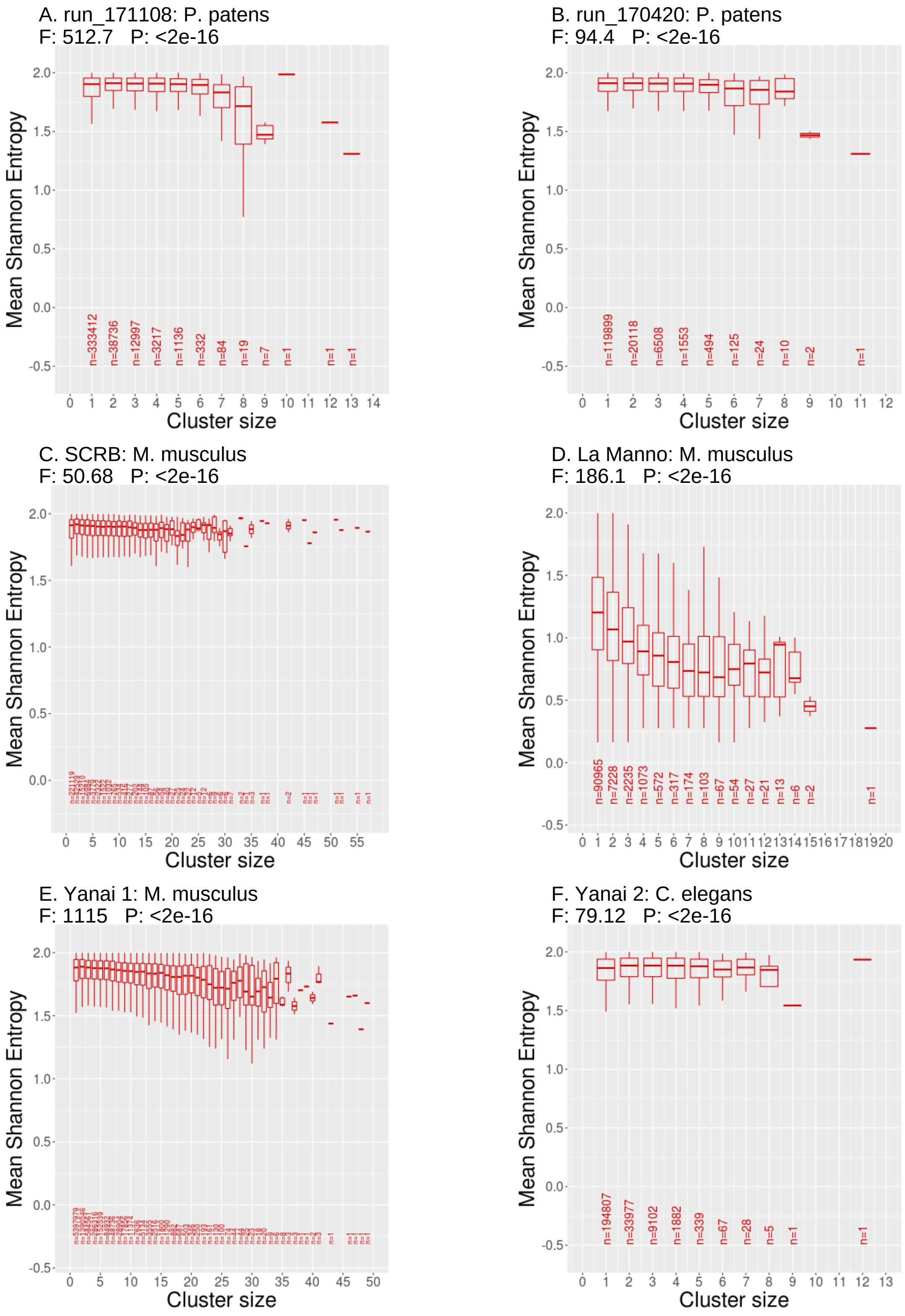

Figure S 7. Shannon Entropies plotted as a function of cluster size for the six data sets.

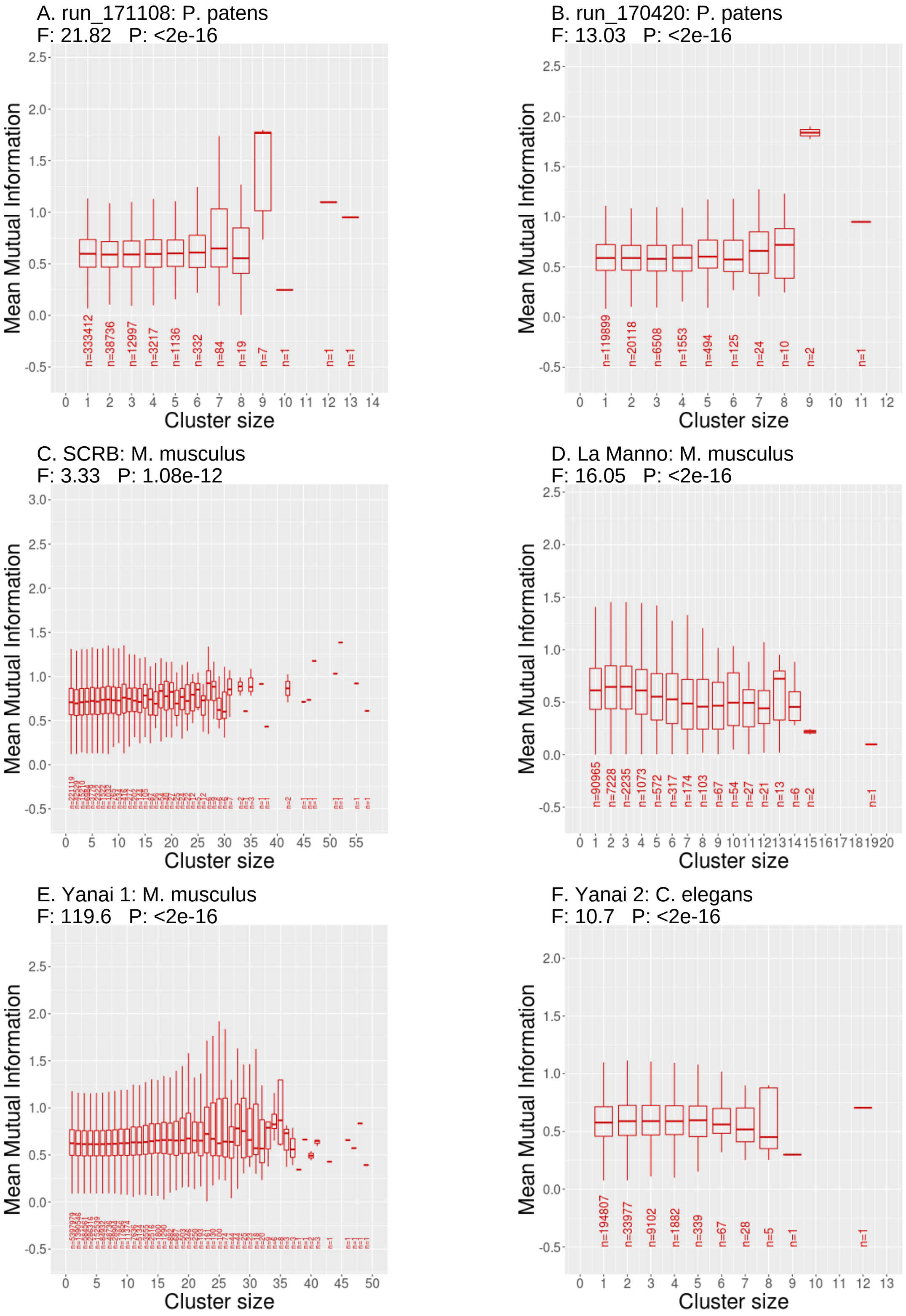

Figure S 8. Mutual Information as a function of cluster size for the six data sets.

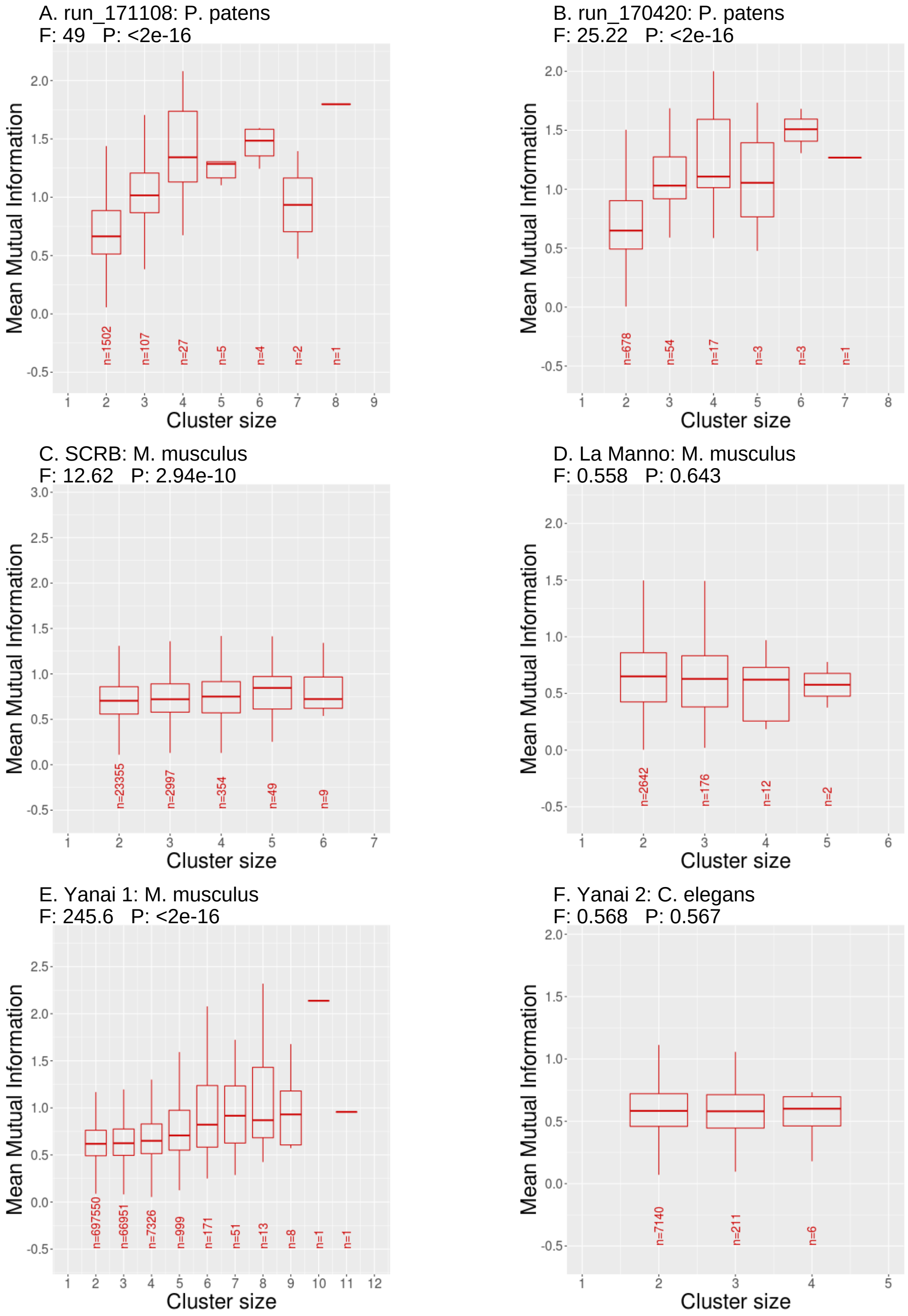

Figure S 9. Mutual Information as a function of cluster size for the six data sets, for reads showing shift sizes of strictly 2.

ACTGACACATTTTTACAGACACAGATCCACACAGAC
ATATATATATATATATATGCACACACAGAACTGTAT
AGCAATAATCTAGTGGTTTCTGATTGAAACAAGTGT
GAGAGAGAGAGAGAGAGAGAGAGATTGGTGTTTGTG
AAGAAATCTCTCCCTCTCTCTCTCTCTCTCTCTCTC
TCTCTCTCTCTCTCTCGTAGTAGTACTCTGAATTTG
GAGAGAGAGAGAGAGTGTGTGTGAGTGTGTGTGTTG
CTCTCTCTCTCTCTCTCTCTCTCAGTTTTGGTTGAT
TACAAACTCGGGATCTTCACACACACACACACACAC
CTCTCTCTCTCTCTCTCGTAGTAGTACTCTGAATTT
CTACATCCGAACACACACACACACTCACACTAACAC
GAGAGAGAGAGAGAGCGAGAGAGAGAGAGAGGGAGG
TCTCTCTCTCTCTCTCCACCTCAACCGATTGTTGTG
TGCTACGATGCAGCAACCGGTACACACACACACTCT
CTGTCTCTGTGTATCACACACTCTCTCTCTCTCTCT
CTGTCTCCTTCTCTCTCTCTCTCTCTCTCTCTCTCT
AGAGAGAGAGAGGCAACGTCGTGATCTGGTCAGGTT
AGAGAGAGAGAGAGAGAGAGTGTGTGTGAGTGTGTG
AGCAACCGGTACACACACACACTCTCAGTGTGCAAG
CTCTCTCTCTCTCTCGTAGTAGTACTCTGAATTTGT
ATGCAGCAACCGGTACACACACACACTCTCACTGTG
CGGCGCACAGTGCATCAAGAATCTCTCTCTCTCTCT
CAAGCGCGAGAGAGCGAGAGAGAGAGAGAGAGAGAG
CTCTCTCTCTCTCTCTCGTAGTAGTACTCTGAATTT
GGACGGGGACAGAGAGAGAGAGAGTGGCATATCCTC
AGAGAGAGAGAGAGAGTGTGTGTGAGTGTGTGTGTT
TGTCTCTGCAGTGTGTGTGTGTGTGAAAATGAAGCT
GGAGAGAGAGAGAGAGAGAGAGGCAACGTCGTGATC
AGAGAGAGAGTGTGTGTGAGTGAGAGATTGAGAGAG
GTGTGTGTATGTGTGAGAGAGAGAGAGAGCAAGAGA
TCTCTCTCTCTCTCTCTCTCAGTTTTGGTTGATGCC
ACTGAGACACCAAGAGAGAGAGAGAGAGAGAGAGCC
CAAGCGCGAGAGAGCGAGAGAGAGAGAGAGAGAGAG
ATCTCTCTCTCTCTCGTAGTAGTACTCTGAATTTGT
TGTGTATCACACACTCTCTCTCTCTCTCTCTCGCTC
TACCACCAGGACTTGCAAACACACACACACACACAC
GAGAGAGAGAGAGAGAGAGAGAGCGAGAACGGAGGG
CTCTCTCTCTCTCGTAGTAGTACTCTGAATTTGTGT
AGGAGGAAAACGGGAGAGAGAGAGAGAGAGAGAGAC

Table S 2. Run_171108 reads found in clusters greater than size 3 with shifts of strictly 2.

GATGCAGCAACCGGTACACACACACACTCTCACTGT
AATCGACCGCAGCGGAGCGAGAGAGAGAGTGAAAGT
AGAGAGAGAGAGAGAGAGAGAGAGAGAGAGATTGGTGT
CTCCTGTCGGCTCTACGAAGAGAGAGAGAGAGAGAC
GGTGTGTGTGTGTGTGTGTGTTTGCTTGCTTGCATG
TGGCATGGCTTCGCCCCCCAAAAAAAAAAGAGAGAG
AGCAATAATCTAGTTGTTTCTGATTGAAACAAGTGT
GAGAGAGAGAGAGAGAGAGAGAGATTGGTGTTTGTG
GAGAGAGAGAGAGAGAGAGAGAGAGAGAGAGTGGTG
TGCAGCAACCGGTACACACACACACTCTCACTGTGC
AATCTCTCCCTCTCTCTCTCTCTCTCTCTCTCTCTC
GATGCAGCAACCGGTACACACACACACTCTCACTGT
ATGCAGCAACCGGTACACACACACACTCCCACTGTG
TGGAGGCAGAGTGAGAGAGAGAGCTAATGAGACTAG
CACAATATCACGAGCACACACACACACACACACACA
GTCGCTCACACACACACACTTCACTTCGATCGCGGC
ATTGTGTGTGTGTGTGTGTGTTGCGCCGCCCTCACC
CTTCACACACACACACACACACAGCAACAACTGCAG
GATGCAGCAACCGGTACACACACACACTCTCACTGT
GCTACGATGCAGCAACCGGTACACACACACACTCTC
CTCTCTCTCTCTCTCTCTCTCTCAGTTTTGGTTGAT
AGAGAGAGAGAGAGAGAGAGAGAGAGAGAGTGGTGT
AGGTATGCCGAGAGAGAGAGAGAGAGAGAGAGAAAC
GATGCAGCAACCGGTACACACACACACTCTCACTGT
ATGGCTTCGCCCCCCAAAAAAAAAAGAGAGAGAGAG
GACAAACTCGGGATCTTCACACACACACACACACAC
AGAGAGAGAGAGAGAGAGAGAGAGATGGGGCAGGGT
CTCTCTCTCTCGTAGTAGTACTCTGAATTTGTGTGC
CTCTCTCTCTCTCTCTCTCGTAGTAGTACTCTGAAT
GAGAGAGAGAGAGAGAGCGAGAGAGAGAGAGAGGGA
TGTGTGTGGTTCGGAGGAGGTGAACAAGCTTGAGAA
GAAGGTATGCCGAGAGAGAGAGAGAGAGAGAGAAAC
TGCTACGATGCAGCAACCGGTACACACACACACTCT
ACATTATAAAAAATAAAAATAATAAAAAAAAAAACA
TCGTAGTAGTACTCTGAATTTGTGTGCTTTGCGGAG
CTGTCTCTGTGTATCACACACTCTCTCTCTCTCTCT
AGAGAGAGAGAGGCAACGTCGTGATCTGGTCAGGTT
AGAGAGAGAGAGAGAGAGAGAGTGTGTGTGAGTGTG
CCGGCTCGATCGTGAAATTTTCAGAAACTCTCTCTC
CTCTCTCTCTCTCTCGTAGTAGCACTCTGAATTTGT

Table S 3. Run_170420 reads found in clusters greater than size 1 with shifts of strictly 2.

GAGGGTGACAAGCACACCCTNAGCAAGAAGGAG
AAAGGCTTTTGGNCTTTTCAATCACTTGCTGAT
CACCATGACAACTCAGTTCTNAAATTCAACTAT
AGGCTCATCACANTTGGATGCAAGCACACAAAG
AGCACACCCTGAGCAAGAAGNAGCTGAAGGAGC
TTATCTATGACTCTGACTGGAACCCCCATAATGA
GACCAAGTATGTGGAAGCCAAGGACTGTCTGAAC
ATTGTTGACTGTCCATAGTCCACGCAGAGTTAC
GACGTTATTCACAGGACGATTCGAAAAGTCCATT
GCAATTCTCCTGNCTCAGCCTCCTGAGTAGGTG
CAGCAACCATGAGTGGTTCCATGATGGGAAGTG
AAATGAATATTATCCCTAATACCTGCCACCCCA
CTTGCTCGCGTTGATCTTGGATCCCTCTGCGAA
GTATGAAATTGGTAGTGGATCATCAAATATAAT
ATCTATCTATCTATCTATATCTATCTATGCATCT
CTGTCAGTATTAAGGCCCAGCAGCCTGTTGATA
GTACATAGTTGACTGACAAANTTCTCTACCATC
CAGAGGGACTCGGAGATCATGCAGCAGAAGCAG
GTTATCGACTACATTGATAGTAAATTTGAGGAC
GAGACACTTGGACGTCACATGTGAAGCCTACGA
CACATCTGCTGGNAGGTGGACAGAGAGGCCAGG
GTTAGTGACAGATTGCTAAACTTGGCTTTAGAC
AGTTAGCTGCAGTAATGGTAGTCTTCCCTCTGG
AATACTTGAACANTTTGCCCAGTAATGTGACAC
ACCTGAAGGTGAAGGGGAATGTGTTCAAAAACA
TGCAATCAGTGAAGAAAAACNCCGTGAGATATT
ACCTGAAGGTGAAGGGGAATGTGTTCAAAAACA
GTGTATGTAGGAGTGAATATAAAAGGACTTCAT
GTACCTGAAGGTGAAGGGGAATGTGTTCAAAAA
GTACCTGAAGGTGAAGGGGAATGTGTTCAAAAA
GTATAATAATTTGAGATGTTNTTAATTATTTTG
CTCTGATTGGCAACATGTTANCTTTGAAGTGGA
TTGTTATGTGAGAAATGTTACTGGGGAAATAGA
TCACCTTCGTGAATACCAAGACCTGCTCAATGTT
GTATGGGAAACAATCTTTTGTAAATGCAAAGCT
GTTTTTGTGTGTGTGTGCTGNCGCTTGTCGACA
CACTGTGTCTCCGACCATTTNCCTTTTCTTTTT
TTCCCAAGCAATTTTGATGGAATCGACATCCACA
TAAATCAATCTGAATGGTATCATTCACCTTGATG
TTCAATCTGTATGTCTGCGAGCTTCACCTTCTGT

Table S 4. SCRB reads found in clusters greater than size 4 with shifts of strictly 2.

```
AAAAAAAAAAAAAAAAAAAAAAAAATCGGGGGGCTGTTTTTT
AAAAAGGCGTGGGTTTTTTTTTTTTTTTTTTTTTTTTTTTTT
AAAAAAAAAAAAAAAAAAAAAAACAGCCGGGGGTTTTTTTTT
AACCCCTCCCATTTTTTTTTTTTTTTTTTTTTTTTTTTTTTT
AAAAAAAAAAAAAAAAAAAAAAAAAAAAAAAAAAGTGGTTTT
AAAAAAAAAAAAAAAAAAAAAAAAAACGCGGGGTGCCCCCTT
AAAAAAAAAAAAAAAAAAAAAAAAAACGGCGTTCTTTTAAAA
AAAGCCCCTCTCTTTTTTTTTTTTTTTTTTTTTTTTTTTTTT
AAAAAAAAAAAAAAAAAAAAAAACCTTCTTTTTAAAACAATT
AAAAAAAAAAAAAAAAAAAAAAAAAAACTAGGGGGGGCCCCC
AAAAAGAAAAAATCTTTTTTTTTTTTTTTTTTTTTTTTTTTT
AAACCGACCTTTTTTTTTTTTTTTTTTTTTTTTTTTTTTTTT
AAAAAAAAAAAAAAAAAAAAAAAAAAGCGGGGGGCCCCCTTT
AAAAAAAAAAAAAAAAAAAAAAACGGTTTTTCTTATACATAT
AAAAAAAAAAAAAAAAAAAAAACCCTCTCTTTTAAAAAAATC
AAAAAAAAAAAAAAAAAAAAAAAACCGTGGCTGGCTGTTTTT
AAAAAAAAAAAAAAAAAAAAAAAGGGCTTTTTTTTTATAAAA
GAACAGCCTGTTTTTTTTTTTTTTTTTTTTTTTTTTTTTTTT
AAGACACAGGTTTTTTTTTTTTTTTTTTTTTTTTTTTTTTTT
AAAAAAAAACTGTTTTTTTTTTTTTTTTTTTTTTTTTTTTTT
AAAAAAAGCCCCCCGATGTTTTTTTTTTTTTTTTTTTTTTTT
AGGGCGGGTTTTTTTTTTTTTTTTTTTTTTTTTTTTTTTTTT
TTTTTTTTTTTTAAGATTTTTTTTTTTTTTTTTTTTTTTTTT
AAAAAAAAAAAAAAAAAAAAAAAAAACGACCGGGTGTTTTTT
AAAAGAAAGTGGTTTTTTTTTTTTTTTTTTTTTTTTTTTTTT
AAAGCGGTTTTTTTTTTTTTTTTTTTTTTTTTTTTTTTTTTT
AAGACAGGGCTTTTTTTTTTTTTTTTTTTTTTTTTTTTTTTT
AAAAAGAGCACCATTTTTTTTTTTTTTTTTTTTTTTTTTTTT
AAAAAAAAAAAAAAAAAAAAAAAAAAAAAAAGCGAGGGGGGC
CCCGTAGTTTTTTTTTTTTTTTTTTTTTTTTTTTTTTTTTTT
AAAAAAAAAAAAAAAAAAAAAAAAAAAAAAAAATGCTTGCAT
AAAAAAAAAAAAAAAAAAAAAAAAAAAAAACAGGTGGGGGGT
AAAAAGCTAGTTTTTTTTTTTTTTTTTTTTTTTTTTTTTTTT
CCAAGTGTTTTTTTTTTTTTTTTTTTTTTTTTTTTTTTTTTT
GCCCCCTCTTTTTTTTTTTTTTTTTTTTTTTTTCTTTTTTTT
AAAAAAAAAAAAAAAAAAAAAAAAAAAAAAAACTTTTCTTTT
ACAGCCGCCATTTTTTTTTTTTTTTTTTTTTTTTTTTTTTTT
AGGGGTATTTTTTTTTTTTTTTTTTTTTTTTTTTTTTTTTTT
CCCCCCCTTTCTTTTTTTTTTTTTTTTTTTTTTTTTTTTTTT
AAAAAAAAAAAAAAAAAAAAAAAAAAAAAAAAAAAACGTCGG
```

Table S 5. La Manno reads found in clusters greater than size 2 with shifts of strictly 2.

AGTGTTCACAAATGATGGGCTGAGGATGAGGGCGG
GGAAGAAAGGACATTGGCGTGAGGACAGTGGGGAG
GTGTGTGTGTGTGTGAGAGAGAATATGTATGTGCG
CTGGGTTCTCTGCTGTTCTTCTGTGTGTGCGTGTG
GTGTGTGTAGTGTGTGTGGTGTATGCATGTGTGTA
TTGCTGGTGGGCCGGAGTGATGTGTTATGGAGGGA
GGCACAGGTGCGTGAGGCCCTGAGGAGTGGAAAGA
CCTGTTCCCAAACTCTCCTCCACACATGCCAACGC
CACACACACACACACACACATGTTAAGTTAAAATT
TTTTTCTTCTCTGTCTGTATACCTCTGTGTGGGTG
GTGTGTGTATTGTTGTTGCAGTTTTCCCTTTGCAT
GTGTGTGTGTGTAAATGGTATAGATGTGGATAAAG
CACACACACACACACACAATTTATTATCTCTGTGA
GTGTGTGTGTGTGTGTGTGTGGACTGAGGCTTTTC
GTTACAGTTTCAGCTGGTAGGGTTTTTGTTGTGTG
TGTATGTAAATTTCAAACCTGTGTGTGTGTGTGTG
TGAGTCTTAGGAAGGTGTGTGTGTGTGTGTGGGTG
TTAACTTACATCACTCCACACACACACACACACAC
GACACACACACACACACAGCAACACATATATAAGA
GCGCTGGGGTGGAGTGCTAGCATTTCAAGAAAAAA
CACACACACACAATTTATTATCTCTGTGAGGAGAG
TGATCTGACAGGTGCTAACATAGTTACACACACAC
TTTTTTGTTTGTTTGTTTCTCTGTGTCTGTGTGTG
TTTCATCCCACCAGGAAACTGTTTTACACACACAC
CACACATTTTTTAAGAAGAGGAATCTGGGCTTTCA
TTGGCCTCATACTCACCATGTACAAACACACACAC
CACACACACACACACACAGGGTCTGGCTCTGTATC
CACACCATGTACTCATAGACCTAAGTGCACCCTCC
CACGCACACACACATACAGTTGCAAACATGCCCAC
ATTCCTTTTGTGTATGTAAATTTCAAACCCGTGTG
TGTGTGTGATGTGTGTGTGTGTGTGATATGTGCGA
CTTCTAAGATGTATTTGTGTGTGTGTGTGTGTGTG
CACACGCGCATACACACACACCCAAACGTACACAC
TCTTTTAAAAAATGTTTCCTTATTGTGTGCGTGTG
ATGAGTCCCTCTCCTGATTATATGTCGTGTGTGTG
CACACACACACACACACACAATTTATTATCTCTGT
CACACACACACACACACGCATACACATACGTGTAC
CACACACACACACACATTTCTAAATATAACCTGTA
GTGTGTGTGTGTGGGGGGGAGATTTTCTGGATTGG
GTGTGTGTGTGTGTGTATACCAGTGCATTAAAATA

Table S 6. Yanai1 reads found in clusters greater than size 6 with shifts of strictly 2.

TTGAGACCGGTTTTACATTACAGCAATCATTGAAC
TTTCTTGGCAAATTGCCAATTCCATACTTTTTCAG
GCAAAAGACACAATTAAACGACCGGGCTTGGGGGA
TGTGCTTTTTTCTACAAACTTTATACATATAACTC
ATGTACCAGCTCATAAGCATACTGTTCATGGCTAG
CATTGTCGCCATCAACAAGGATCCTGATGCACCAA
GGTTGACAAGCAAGACAAGAAAAAGAAGAAGCGCG
TATGGTACAACATGAAACATTGATCGCATCGAGAC
TAAAAACCACCAAAAACCGCGGAGCGACTTGACGC
CGTGATTACCCGCTGAACTTAAGCATATCATTTAG
AAAACAATCAATTAACCCGAGAGAATAGGAGAGAC
AAGTCTCCCGAATATTTTTATTGATTTATTGTTTA
CCCTCCATGAGATTGTTTTTATGTATGAGTAGATA
TCCAACATTTGGATCCTCGGTGACTTGTTGTGGCG
TTCCAATCATTTTTCCAATATCCCAAAGCTGGACT
AAGCCCATGTCGTCGTCGGATTCCTCCTTTGGCTC
ATTATGCTTTTCAAAAAAATCGATCGTTGGTTCTT
TTCCGTTGAATTTGACGTGATCTCCTGCTTGTCCC
CCTCTCCAAAAATCCAAGTTTGTGGAGCGAAATGC
GTGACGCTCGCTGAAGTTGTTGTTGTTGACGGTCT
TAGAATTACGAGGAGACGAAAAGGCACGACAACCC
TAAATACATTCCAGATTCAACTTGTAGTATTATAT
GCTCAGTCGTGATTACCCGCTGAACTTAAGCATAT
TTGGGTTTCCATTAGAAGTATGAAATTTCCATTCA
TGAATAAAATAGTTATACCGGGTACGGCGTTTTTG
ATTTTTTGAAGCGAAAAAAACTAGGGAAAATATAG
ATGTCCTCGATCTTTTTTTTAACGCAAAATTTTTT
CAGCAATTGAAAAAAACAAACAAAGAAAAAATAAA
TGTTGAATCCGGCGTTCTTGGCCAAGCGACGGACT
CCATCATCACGGACCACCACCACCACCATACTACG
AGCATCTGCCCCAGCTGCCGTTGAAGCCGCTCCAG
ACACCGTTATGGTGCTTCCCTCCGTAAGATGGCCA
ACTCGGATGGGGAGTTGATAATGGAACACCATATT
AAACGTCTCCGCAGCTCAAATGCACTATCAAATCT
ATCTCAACCTGAACTCAGTCGTGATTACCCGCTGA
CCAAAAATCCTTTAAAAACTGTTATGTTAATATGT
CTCTCAACCTGAACTCAGTCGTGATTACCCGCTGA
TCTCTCCAGCTTCTCCCTCAGCGTATCCGTCGACG
ATAATCCATTCCATAATCTGCTTCACGGAATAACT
TTCTGTCAATTTGATCGCTTTAACGTCGGATACTT

Table S 7. Yanai2 reads found in clusters greater than size 1 with shifts of strictly 2.

Gene ID	Gene Name	Gene ID	Gene Name
gene15014	Rtkn	gene37995	Arhgap31
gene40749	Usp14	gene29487	Gm9548
gene32668	Gm3604	gene3375	Ralgps1
gene12461	Lrrc8c	gene34036	Pbrm1
gene13363	Slc29a4	gene32584	Ntrk2
gene37400	Clec16a	gene1057	Cab39
gene109	Tram1	gene1513	Nckap5
gene4514	Lrrc4c	gene27131	Thg1l
gene35044	Ints9	gene17187	Lipe
gene16653	Slc27a5	gene9456	Focad
gene33282	Cdk7	gene22590	Ntm
gene31570	Arid4b	gene26390	R3hdm2
gene1248	Kif1a	gene37153	Slc4a8
gene27317	Gm30366	gene43021	Smc3
gene25570	Rnf126	gene11952	Gabrb1
gene2796	Gm13219	gene23508	Adam10
gene6164	Arfgap1	gene30724	Foxn3
gene3319	Lamc3	gene21163	Arhgap10
gene1742	Nek7	gene17186	4732471J01Rik
gene11721	Gm16223	gene35684	Plcxd3
gene204	Kcnq5	gene25168	Mcu
gene25858	Chpt1	gene29317	Eif4a3
gene13943	Podxl	gene13869	Snd1
gene26601	Tug1	gene30450	Synj2bp
gene11162	Abcb4	gene16205	Tmtc1
gene25938	Gm32749	gene33218	Arhgef28
gene6764	Gm32177	gene19435	Scnn1g
gene32038	Cdkal1	gene7999	Mettl14
gene25857	Sycp3	gene12037	C530008M17Rik
gene21868	Nudt7	gene25083	Dcbld1

Table S 8. Mouse genes in the Figure S4 data having apparent overexpression with adjusted $P < 0.05$

Accession	Tissue	Sample Barcode	Run	Sequencing Lane
SRR7295917	Whole Plant	ACATCG	171108	1
SRR7295918	Whole Plant	ATTGGC	171108	1
SRR7295919	Whole Plant	CACTGT	171108	1
SRR7295920	Whole Plant	GATCTG	171108	1
SRR7295921	Whole Plant	TACAAG	171108	1
SRR7295922	Whole Plant	TGGTCA	171108	1
SRR7295923	Whole Plant	ATTGGC	171108	2
SRR7295924	Whole Plant	CACTGT	171108	2
SRR7295925	Whole Plant	GATCTG	171108	2
SRR7295926	Whole Plant	TACAAG	171108	2
SRR7295931	Whole Plant	TGGTCA	171108	2
SRR7295932	Whole Plant	ACATCG	171108	3
SRR7295933	Whole Plant	ATTGGC	171108	3
SRR7295934	Whole Plant	CACTGT	171108	3
SRR7295927	Whole Plant	GATCTG	171108	3
SRR7295928	Whole Plant	TACAAG	171108	3
SRR7295929	Whole Plant	TGGTCA	171108	3
SRR7295930	Single Protoplast	ACATCG	171108	4
SRR7295935	Single Protoplast	ATTGGC	171108	4
SRR7295936	Single Protoplast	CACTGT	171108	4
SRR7295908	Single Protoplast	GATCTG	171108	4
SRR7295907	Single Protoplast	TGGTCA	171108	4
SRR7295910	Whole Plant	ACATCG	171108	6
SRR7295909	Whole Plant	ATTGGC	171108	6
SRR7295912	Whole Plant	CACTGT	171108	6
SRR7295911	Whole Plant	GATCTG	171108	6
SRR7295914	Whole Plant	TACAAG	171108	6
SRR7295913	Whole Plant	TGGTCA	171108	6
SRR7295916	Whole Plant	ACATCG	171108	7
SRR7295915	Whole Plant	CACTGT	171108	7
SRR7295939	Whole Plant	TGGTCA	171108	7
SRR7295940	Single Protoplast	ACATCG	171108	8
SRR7295937	Whole Plant	CACTGT	171108	8
SRR7295938	Single Protoplast	GATCTG	171108	8
SRR7295905	Single Protoplast	TACAAG	171108	8
SRR7295906	Single Protoplast	TGGTCA	171108	8
SRR7295941	Whole Plant	NA	170420	4

Table S 9. SRA Study (SRP150352) Information for run_171108 and run_170420

Supplementary References

1. Barnett, D. W., Garrison, E. K., Quinlan, A. R., Strömberg, M. P. & Marth, G. T. BamTools: a C++ API and toolkit for analyzing and managing BAM files. *Bioinforma. (Oxford, England)* **27**, 1691–1692 (2011). DOI 10.1093/bioinformatics/btr174.

2. Quinlan, A. R. & Hall, I. M. BEDTools: a flexible suite of utilities for comparing genomic features. *Bioinforma.* **26**, 841–842 (2010). URL https://academic.oup.com/bioinformatics/article/26/6/841/244688. DOI 10.1093/bioinformatics/btq033.

3. Wu, T. D. & Nacu, S. Fast and SNP-tolerant detection of complex variants and splicing in short reads. *Bioinforma. (Oxford, England)* **26**, 873–881 (2010). DOI 10.1093/bioinformatics/btq057.

4. Kim, D., Langmead, B. & Salzberg, S. L. HISAT: a fast spliced aligner with low memory requirements. *Nat. Methods* **12**, 357–360 (2015). URL https://www.nature.com/articles/nmeth.3317. DOI 10.1038/nmeth.3317.

5. Tange, O. Gnu parallel - the command-line power tool. *;login: The USENIX Mag.* **36**, 42–47 (2011). URL http://www.gnu.org/s/parallel.

6. Li, H. *et al.* The Sequence Alignment/Map format and SAMtools. *Bioinforma. (Oxford, England)* **25**, 2078–2079 (2009). DOI 10.1093/bioinformatics/btp352.

7. Dobin, A. *et al.* STAR: ultrafast universal RNA-seq aligner. *Bioinforma. (Oxford, England)* **29**, 15–21 (2013). DOI 10.1093/bioinformatics/bts635.

8. Danecek, P. *et al.* The variant call format and VCFtools. *Bioinforma.* **27**, 2156–2158 (2011). URL https://academic.oup.com/bioinformatics/article/27/15/2156/402296. DOI 10.1093/bioinformatics/btr330.

9. R Core Team. *R: A Language and Environment for Statistical Computing*. R Foundation for Statistical Computing, Vienna, Austria (2017). URL https://www.R-project.org/.

10. Love, M. I., Huber, W. & Anders, S. Moderated estimation of fold change and dispersion for RNA-seq data with DESeq2. *Genome Biol.* **15**, 550 (2014). URL https://doi.org/10.1186/s13059-014-0550-8. DOI 10.1186/s13059-014-0550-8.

11. Wickham, H. *ggplot2: Elegant Graphics for Data Analysis* (Springer-Verlag New York, 2009). URL http://ggplot2.org.

12. Wickham, H., Francois, R., Henry, L. & Müller, K. *dplyr: A Grammar of Data Manipulation* (2017). URL https://CRAN.R-project.org/package=dplyr. R package version 0.7.4.
